# Associations of dietary patterns and longitudinal brain-volume change in Japanese community-dwelling adults: results from the national institute for longevity sciences-longitudinal study of aging

**DOI:** 10.1186/s12937-024-00935-3

**Published:** 2024-03-12

**Authors:** Shu Zhang, Giovanni Sala, Akinori Nakamura, Takashi Kato, Kanae Furuya, Hiroshi Shimokata, Xiang Gao, Yukiko Nishita, Rei Otsuka

**Affiliations:** 1 Department of Epidemiology of Aging, National Center for Geriatrics and Gerontology, 7-430 Morioka-cho, Obu, 474-8511 Aichi Japan; 2 Department of Psychology, University of Liverpool, Liverpool, Merseyside UK; 3 Department of Biomarker Research, National Center for Geriatrics and Gerontology, Obu, Aichi Japan; 4 Department of Clinical and Experimental Neuroimaging, National Center for Geriatrics and Gerontology, Obu, Aichi Japan; 5 Graduate School of Nutritional Sciences, Nagoya University of Arts and Sciences, Nisshin, Aichi Japan; 6 Department of Nutrition and Food Hygiene, Fudan University, Shanghai, People’s Republic of China

**Keywords:** Longitudinal study, Brain atrophy, Community-dweller, Dietary pattern, Japanese

## Abstract

**Background:**

The association of dietary patterns and longitudinal changes in brain volume has rarely been investigated in Japanese individuals. We prospectively investigated this association in middle-aged and older Japanese community-dwelling adults.

**Methods:**

Data with a 2-year follow-up from the sixth wave (July 2008 to July 2010; baseline) to the seventh (July 2010 to July 2012; follow-up) of the National Institute for Longevity Sciences-Longitudinal Study of Aging project were analyzed. Dietary intake was assessed using a 3-day dietary record, and longitudinal volume changes (%) in the total gray matter (TGM), total white matter, and frontal, parietal, occipital, temporal, and insular lobes were assessed using 3-dimensional T1 magnetic resonance imaging scans. Multiple factor analysis and hierarchical clustering revealed sex-specific dietary patterns. Associations between dietary patterns and annual brain-volume changes (%) were evaluated using general linear models adjusted for age, apoprotein E genotype, body mass index, medical history, lifestyle behaviors, socioeconomic factors, and energy intake.

**Results:**

Among the 1636 participants (age: 40.3–89.2 years), three dietary patterns were determined for men (*n* = 815; Western; Vegetable-Fruit-Dairy; and Traditional Japanese diets) and women (*n* = 821; Western; Grain-Vegetable-Fruit; and Traditional Japanese diets). Compared to women following the Western diet, those on the Traditional Japanese diet had less TGM atrophy. Multivariable-adjusted *β* (95% confidence interval) of the annual change (%) of TGM was − 0.145 (-0.287 to -0.002; *P* = 0.047), which correlated with reduced parietal lobe atrophy. No association between dietary pattern and brain atrophy was observed in men.

**Conclusions:**

Adherence to healthy dietary patterns, with higher consumption of whole grains, seafood, vegetables, fruits, mushrooms, soybean products, and green tea, potentially confers a protective effect against brain atrophy in middle-aged and older Japanese women but not in men. Further research to confirm these results and ascertain the underlying mechanisms is required. This study highlights the importance of sex-specific effects on the relationship between dietary patterns and brain health in diverse populations.

**Supplementary Information:**

The online version contains supplementary material available at 10.1186/s12937-024-00935-3.

## Background

Healthy dietary patterns constitute modifiable lifestyle behaviors that play a crucial role in promoting longevity and healthy aging. The significant efficacy of the Mediterranean diet (MeDi), a widely recognized dietary pattern, in preventing cognitive decline and dementia risk has been demonstrated [1]. Age-related brain atrophy is a contributory factor in the pathogenesis of cognitive decline and dementia [2, 3]. Advances in magnetic resonance imaging (MRI) technology have resulted in an increasing research focus on the relationship between dietary patterns and longitudinal brain-volume changes to explore and elucidate the positive effects of dietary patterns on brain health and their potential to delay the aging process.

The beneficial effects of the MeDi in preventing brain atrophy have been demonstrated [4], and cross-sectional studies have shown that, among older adults, adherence to the MeDi is associated with larger brain volume [5], greater cortical thickness [6, 7], and better white matter (WM) microstructural integrity [8]. A longitudinal cohort study conducted in older Scottish individuals showed that lower adherence to the MeDi predicted total brain atrophy during a 3-year period [9]. A randomized controlled trial indicated the neuroprotective effects of a high-polyphenol MeDi against age-related brain atrophy [10]. However, the MeDi does not align with Asian dietary habits, and the findings observed in Western populations may be difficult to generalize to Asian populations. Therefore, we hypothesize that there are one or several dietary patterns suitable for Asians, and adhering to these dietary patterns will have a positive impact on preventing brain atrophy in older adults, similar to the beneficial effects observed in Western populations following the MeDi.

This study was conducted to investigate whether the major dietary patterns observed in Japanese adults are associated with an altered risk of brain atrophy in a large Japanese cohort.

## Methods

### Study cohort

Data analyzed in the present study were collected as a part of the National Institute for Longevity Sciences-Longitudinal Study of Aging (NILS-LSA) project – a Japanese population-based prospective cohort study of normal aging and age-related diseases. Participants were recruited via age- and sex-stratified random sampling from Obu and Higashiura Town in Aichi Prefecture, Japan. The first-wave examination of the NILS-LSA was conducted from November 1997 to April 2000 and included 2267 participants (age 40–79 years). These participants were followed up every 2 years and replaced by new, randomly recruited, age- and sex-matched participants when the original participants (age 40–79 years) could no longer attend the follow-up investigations. Participants aged 40 years were recruited annually. Details of the NILS-LSA have been described previously [11]. This research involving human participants strictly adhered to the principles outlined in the Declaration of Helsinki. Approval for conducting the study was obtained from the Committee on the Ethics of Human Research at the National Center for Geriatrics and Gerontology (Approval No. 1665-2) before the commencement of data collection. All participants provided written informed consent for data collection and analysis before participating in the study.

Participants of this study were selected from the sixth (July 2008 to July 2010) and seventh (July 2010 to July 2012) waves of the NILS-LSA because 3-dimensional T1 MRI data were acquired from these two waves. In this study, the sixth wave was used as the baseline and the seventh wave was used as the follow-up examination. Of the 2302 individuals who participated in the baseline survey, we excluded 315 potential participants who did not participate in the follow-up, 78 who did not undergo MRI because of claustrophobia or other reasons or had defective MRI data at baseline or follow-up, 76 whose FreeSurfer estimation failed, 4 with a self-reported history of dementia, 13 with a history of head surgery at baseline or follow-up, 2 whose MRI images showed apparent new cerebrovascular lesions diagnosed by a radiologist at the follow-up, 113 whose nutritional assessment data were incomplete at baseline, 32 with a Mini-Mental State Examination (MMSE) score < 24 at baseline, 6 whose apoprotein E (APOE) genotype data were unavailable, and 27 whose energy intake (kcal/day) was < 500 or > 3000 at baseline. Thus, 1636 Japanese individuals (815 men and 821 women, age 40.3–89.2 years) were included as participants, and their data were analyzed in this study.

### Dietary intake assessment

At baseline, dietary intake was assessed using a 3-day dietary record, which was completed over three continuous days (two weekdays and one weekend day) [12], and the average daily consumption was calculated. Most participants completed the dietary records at home and returned the completed records within 1 month. Participants recorded the volume of beverages (in mL) or weighed the foods (in g) using 1-kg kitchen scales (Sekisui Jushi, Tokyo, Japan) and used a disposable camera (27 shots; Fuji Film, Tokyo, Japan) to take photos of their meal plates before and after meals. Dietitians used these photos to obtain complete data and contacted the participants via telephone to resolve any discrepancies or obtain further information as necessary. Energy intake (kcal/day) was calculated according to the Standard Tables of Food Composition in Japan (STFCJ, 2010) and other sources [12, 13].

### Magnetic resonance imaging

The MRI scans at baseline and follow-up were performed using the same 3.0-Tesla MRI scanner (Siemens Magnetom Tim Trio, Erlangen, Germany) with the Magnetization-Prepared Rapid Gradient-Echo Imaging sequence [14]. High-resolution 3D T1-weighted images were acquired (TR/TE/TI = 1800/1.98/800 ms, 9-degree flip angle, 0.98 × 0.98 × 1.1 mm^3^ resolution, and 256 × 256 matrix). FreeSurfer version 5.3 (http://freesurfer.net) [15] was used, in accordance with the previously described technical details [16], for cortical surface reconstruction and estimation of regional gray matter (GM) and WM volumes. The fully automated procedure in FreeSurfer involves preprocessing the participant’s image data, segmenting the cortical GM and WM, tessellating the GM/WM junction, inflating the folded-surface tesselation patterns, and automatically correcting topologic defects. Subsequently, FreeSurfer parcellated the cerebral cortex into gyral-based regions of interest (ROIs) according to the Desikan–Killiany atlas [17, 18] and performed automatic subcortical segmentation [19]. To minimize the confounding effect of interindividual morphological variability, a longitudinal stream for FreeSurfer was used [20]. If any of the FreeSurfer processes, such as segmentation, failed, the data from these cases were excluded from the analysis.

Furthermore, considering interindividual differences in cranial size, the relative volume (normalized by intracranial volume) of the total gray matter (TGM) and total white matter (TWM) was calculated separately, at baseline and follow-up, using the following formula:

$$\text{Relative volume of the ROI = ROI volume (mm}^3\text{)/total intracranial volume (mm}^3\text{)}$$

To confirm the effect of dietary patterns on specific brain lobes, the same methodology was used to derive relative volumes of the frontal, parietal, occipital, temporal, and insular lobes; the mapping of individual “Desikan–Killiany” ROIs was defined with reference to a previous study [21].

### Other measurements

In the baseline survey, data on medical history (i.e., stroke, hypertension, heart disease, dyslipidemia, and diabetes; dichotomous yes or no responses, for each), smoking status (never, former, or current), and education level (≤ 9, 10 − 12, or ≥ 13 years) were collected using a self-administered questionnaire. Body mass index (BMI; kg/m^2^) was calculated from data collected in the baseline survey using the following formula: BMI = weight (kg)/height^2^ (m^2^). Height and weight were measured to the nearest 0.1 cm and 0.1 kg, respectively, using digital scales in a fasted state (around 9–10 a.m.) with participants wearing light clothing and no shoes. The 24-hour total physical activity was assessed using the metabolic equivalent of task (MET) score (METs-h/day; continuous), which was obtained using a semiquantitative assessment of data from participant interviews that were conducted by trained interviewers [20]. Depressive symptoms were assessed by the Center for Epidemiologic Studies Depression (CES-D) Scale [22, 23] (where a CES-D score ≤ 15 or ≥ 16 indicated normal status or the presence of relevant depressive symptoms, respectively). Among participants aged ≥ 60 years (818 participants), the Mini-Mental State Examination (MMSE) [24] (Japanese version) [25] score was assessed at baseline based on participant interviews conducted by trained clinical psychologists or graduate students who were majoring in psychology. Genomic DNA was extracted from the peripheral blood lymphocytes using standard procedures. APOE genotypes were determined using polymerase chain reaction amplification [26].

### Statistical analysis

All analyses were performed separately for men and women. A two-step approach was employed to derive dietary patterns. First, multiple factor analysis (MFA) – a type of weighted principal component analysis (PCA) – was used to estimate the dimensions of individual food items (dimensionality reduction). The dimensions represent groups of correlated food items – that is, foods that are likely to be consumed together (e.g., as in common meals). Thereafter, the derived dimensions were used to obtain dietary patterns (clusters) by using hierarchical clustering on principal components (HCPC); a minimum of three dietary patterns was set, and a significance threshold of 0.05 was used to select dimensions that characterized the dietary patterns. The entire procedure has been described previously [27].

An advantage of using MFA (rather than standard PCA) in combination with HCPC is that MFA jointly calculates the importance of both individual food items as well as prespecified food groups (e.g., spinach and carrots as yellow–green vegetables) in the estimation of dietary patterns (clusters). Besides providing a more accurate description of the participants’ dietary patterns, this approach also eliminated the need to sum up the consumption of similar foods (e.g., the sum of all green-vegetable items consumed), which is often arbitrary and results in the loss of valuable information.

In the NILS-LSA 3-day dietary record survey, a total of 1998 codes for foods, beverages, and ingredients were collected. Of these, 1878 items were sourced from the STFCJ (2010), with an additional 120 items not part of the STFCJ (2010). In our analysis, we disaggregated processed foods into multiple single items and excluded items with a mean consumption of less than 1 g/day by the study participants. Consequently, the number of foods, beverages, and ingredients used in the analysis was 231 for men and 239 for women. In the MFA, these foods, beverages, and ingredients were assigned to 21 groups (i.e., “cereals”, “fish & shellfish”, “red meat”, “white meat”, “eggs”, “soybean & soybean products”, “vegetables”, “pickles”, “tubers”, “mushroom”, “seaweeds”, “fruits”, “dairy & dairy products”, “vegetable oils”, “seasonings”, “sweets, sugars & sweeteners”, “green tea”, “other tea”, “coffee”, “soft drinks”, and “alcohol”) based on their nutritional characteristics (without summing up their consumption quantities).

Regarding annual changes in (regional) brain volumes, the follow-up time (in days) for each individual was calculated by subtracting the date of the sixth MRI from that of the seventh MRI. The annual changes in the ROI (%) for the above-defined structures were calculated as follows:

$$\frac{\text{relative volume of ROI}_\text{at baseline}-\text{relative volume of ROI}_\text{at follow-up}}{\text{relative volume of ROI}_\text{at baseline}}\div\frac{\text{follow-up time (days)}}{365.25}\times100\%.$$

The associations of dietary patterns and annual changes in the relative volumes of the TGM, TWM, and other brain lobes were evaluated using a general linear model. Several studies have previously reported differences in the rates of brain atrophy between individuals who have the apoprotein E-ε4 (APOE-ε4) allele and those who lack the allele [28–31]. Therefore, to account for potential confounding factors, Model 1 was adjusted for baseline information, including age, APOE genotype, education level, lifestyle behaviors (i.e., smoking status and total physical activity), and energy intake. Model 2 was further adjusted for BMI, depressive symptoms, and medical history (i.e., stroke, hypertension, heart disease, dyslipidemia, and diabetes).

All statistical analyses described herein were two-sided, and a *P*-value < 0.05 was considered significant. Statistical analyses were conducted using R software (version 4.3.1; R Foundation for Statistical Computing, Vienna, Austria) and RStudio software (version 2023.06.0 + 421).

## Results

### Baseline characteristics of participants with different sex-specific dietary patterns

The mean (standard deviation) follow-up duration was 2.0 (0.1) years for all participants. Overall, three dietary patterns were obtained for men (Additional files Tables 1, 2, 3, 4, 5 and 6) and women (Additional files Tables 7, 8, 9, 10, 11 and 12). Based on their characteristics, the three dietary patterns identified were categorized as the Western diet, Vegetable-Fruit-Dairy diet, and the Traditional Japanese diet in men, and the Western diet, Grain-Vegetable-Fruit diet, and the Traditional Japanese diet in women.

**Table 1:** Baseline characteristics by dietary patterns in both men and women (*n* = 1636)

Men (*n* =815)	Total participants	Western diet	Vegetable-Fruit-Dairy diet	Traditional Japanese diet	*P*-value ^a^
No. of participants	815	310	206	299	
Age (years); mean (SD)	60.8 (11.7)	53.3 (9.0)	65.4 (10.9)	65.4 (10.7)	< 0.001
APOE-ε4 carriers ^b^; %	20.1	21.9	20.4	18.1	0.488
BMI (kg/m^2^); mean (SD)	23.1 (2.7)	23.5 (2.7)	22.7 (2.6)	22.9 (2.6)	< 0.001
Medical history (yes); %					
Stroke	3.4	1.6	4.4	4.7	0.080
Hypertension	30.6	24.8	30.6	36.5	0.008
Heart disease	3.3	1.6	4.4	4.3	0.105
Dyslipidemia	18.7	15.5	18.4	22.1	0.113
Diabetes	8.7	4.5	12.6	10.4	0.003
Current smoker; %	21.0	32.3	17.5	11.7	< 0.001
Total physical activity (METs-h/day); mean (SD)	34.2 (3.8)	34.9 (3.9)	34.0 (4.1)	33.6 (3.3)	< 0.001
Education level (years); %					
≤9	14.0	6.5	18.9	18.4	< 0.001
10−12	36.2	37.1	38.3	33.8	
≥13	49.8	56.5	42.7	47.8	
Depressive symptoms ^c^; %	9.4	7.7	10.7	10.4	0.424
Energy intake (kcal/day); mean (SD)	2185.0 (355.0)	2225.0 (367.0)	2099.9 (341.4)	2201.1 (342.2)	< 0.001
Women (*n* =821)	Total participants	Western diet	Grain-Vegetable-Fruit diet	Traditional Japanese diet	*P*-value ^a^
No. of participants	821	387	144	290	
Age (years); mean (SD)	59.8 (11.9)	55.2 (10.7)	63.0 (10.8)	64.4 (11.9)	< 0.001
APOE-ε4 carriers ^b^; %	19.5	19.9	17.4	20.0	0.777
BMI (kg/m^2^); mean (SD)	22.2 (3.3)	22.2 (3.4)	22.6 (3.3)	22.0 (3.1)	0.162
Medical history (yes); %					
Stroke	2.8	1.8	2.8	4.1	0.181
Hypertension	24.0	18.9	26.4	29.7	0.004
Heart disease	2.7	1.6	2.8	4.1	0.113
Dyslipidemia	21.8	17.6	20.8	27.9	0.005
Diabetes	4.9	3.9	4.9	6.2	0.378
Current smoker; %	4.6	5.9	6.9	1.7	0.012
Total physical activity (METs-h/day); mean (SD)	35.7 (2.7)	35.9 (2.7)	35.8 (2.6)	35.3 (2.7)	0.007
Education level (years); %					
≤9	17.4	12.1	21.5	22.4	0.002
10−12	43.7	44.2	40.3	44.8	
s≥13	38.9	43.7	38.2	32.8	
Depressive symptoms ^c^; %	11.9	11.6	13.2	11.7	0.876
Energy intake (kcal/day); mean (SD)	1819.0 (306.0)	1798.0 (300.9)	1885.6 (309.4)	1813.4 (308.8)	0.013

*SD* Standard deviation, *APOE *Apoprotein E, *BMI *Body mass index, *MET *Metabolic equivalents

^a^For continuous variables, the general linear model was used; for categorical variables, the χ^2^ test and Fisher’s Exact Test were used

^b^APOE genotype was defined as APOE-ε4 carriers (2/4, 3/4, 4/4) and APOE-ε4 noncarriers (2/2, 2/3, 3/3)

^c^Defined by the Center for Epidemiologic Studies Depression Scale (CES-D) score ≥ 16

The baseline characteristics of the participants according to their dietary patterns are shown in Table 1. In men, among the different dietary categories in the order of the Western diet, Vegetable-Fruit-Dairy diet, and the Traditional Japanese diet, we observed a higher proportion of individuals with hypertension, a declining trend in the level of total physical activity, and a reduction in the proportion of current smokers. In women, following the order of the Western diet, Grain-Vegetable-Fruit diet, and the Traditional Japanese diet, an increasing trend was observed in terms of age and proportion of individuals with a history of hypertension and dyslipidemia. Furthermore, in women, total physical activity level and the proportion of individuals with ≥ 13 years of education showed a declining trend across the different groups.

### Sex-specific dietary patterns and brain atrophy

Table 2 displays the relative volumes of TGM, TWM, and other brain lobes at baseline and the annual changes in these relative volumes (%) stratified by dietary patterns in both men and women. The mean annual atrophy rates for the TGM in men were 0.460%, 0.491%, and 0.480% for those on the Western diet, Vegetable-Fruit-Dairy diet, and Traditional Japanese diet, respectively, whereas those in women were 0.299%, 0.355%, and 0.258% for those on the Western diet, Grain-Vegetable-Fruit diet, and Traditional Japanese diet, respectively. Similarly, the corresponding mean annual atrophy rates for the TWM were 0.116%, 0.275%, and 0.408% in men and 0.182%, 0.184%, and 0.360% in women, respectively.

**Table 2:** Relative volumes of total gray matter, total white matter, and other brain lobes at baseline and annual changes in these relative volumes (%) by dietary patterns in both men and women (*n* = 1636) ^a, b^

Relative volume of ROI; mean (SD)	Baseline	Annual changes (%)	Baseline	Annual changes (%)	Baseline	Annual changes (%)	Baseline	Annual changes (%)
Men			Western diet		Vegetable-Fruit-Dairy diet		Traditional Japanese diet	
No. of participants	815		310		206		299	
Total gray matter	0.401 (0.027)	0.475 (0.923)	0.412 (0.023)	0.460 (0.946)	0.395 (0.028)	0.491 (0.962)	0.394 (0.028)	0.480 (0.872)
Total white matter	0.335 (0.022)	0.264 (0.823)	0.341 (0.020)	0.116 (0.732)	0.331 (0.024)	0.275 (0.805)	0.332 (0.022)	0.408 (0.897)
Brain lobes								
Frontal lobe	0.105 (0.008)	0.447 (1.284)	0.107 (0.007)	0.480 (1.360)	0.104 (0.008)	0.523 (1.270)	0.103 (0.008)	0.361 (1.210)
Parietal lobe	0.071 (0.006)	0.545 (1.312)	0.073 (0.005)	0.543 (1.370)	0.070 (0.005)	0.610 (1.320)	0.069 (0.006)	0.500 (1.240)
Occipital lobe	0.030 (0.003)	0.586 (1.226)	0.031 (0.003)	0.495 (1.210)	0.029 (0.003)	0.593 (1.220)	0.029 (0.003)	0.676 (1.250)
Temporal lobe	0.069 (0.006)	0.635 (1.185)	0.071 (0.005)	0.557 (1.210)	0.068 (0.005)	0.645 (1.200)	0.068 (0.006)	0.709 (1.150)
Insular lobe	0.009 (0.001)	0.394 (1.085)	0.009 (0.001)	0.320 (1.050)	0.009 (0.001)	0.427 (1.170)	0.009 (0.001)	0.450 (1.060)
Women			Western diet		Grain-Vegetable-Fruit diet		Traditional Japanese diet	
No. of participants	821		387		144		290	
Total gray matter	0.411 (0.022)	0.294 (0.878)	0.414 (0.021)	0.299 (0.810)	0.406 (0.021)	0.355 (1.000)	0.409 (0.024)	0.258 (0.902)
Total white matter	0.337 (0.020)	0.245 (0.744)	0.338 (0.019)	0.182 (0.654)	0.335 (0.018)	0.184 (0.853)	0.337 (0.021)	0.360 (0.786)
Brain lobes								
Frontal lobe	0.108 (0.006)	0.205 (1.282)	0.109 (0.006)	0.254 (1.200)	0.107 (0.006)	0.281 (1.500)	0.107 (0.007)	0.103 (1.270)
Parietal lobe	0.073 (0.005)	0.346 (1.254)	0.074 (0.005)	0.382 (1.190)	0.072 (0.005)	0.419 (1.440)	0.072 (0.005)	0.263 (1.240)
Occipital lobe	0.030 (0.003)	0.391 (1.231)	0.030 (0.003)	0.346 (1.250)	0.030 (0.003)	0.509 (1.110)	0.030 (0.003)	0.394 (1.260)
Temporal lobe	0.071 (0.005)	0.443 (1.107)	0.072 (0.004)	0.431 (0.947)	0.070 (0.005)	0.481 (1.330)	0.070 (0.005)	0.442 (1.190)
Insular lobe	0.009 (0.001)	0.261 (1.065)	0.009 (0.001)	0.226 (1.020)	0.009 (0.001)	0.445 (0.992)	0.009 (0.001)	0.216 (1.160)

^a^Relative volume of region of interest (ROI) = ROI volume (mm^3^)/total intracranial volume (mm^3^)

^b^ $$\text{Annual changes in region of interest (ROI)}\, (\%)=\frac{\text{relative volume of ROI}_\text{at baseline}-\text{relative volume of ROI}_\text{at follow-up}}{\text{relative volume of ROI}_\text{at baseline}}\div\frac{\text{follow-up time (days)}}{365.25}\times100\%$$

 Among men, compared to those who adhered to the Western diet, none of the dietary patterns were associated with the volume of TGM and TWG at baseline (Additional file Table 13) nor TGM and TWM atrophy (i.e., annual changes in relative volumes) (Table 3). Among women, participants who adhered to the Traditional Japanese diet exhibited less TGM atrophy as compared to individuals who adhered to the Western diet (multivariable-adjusted *β* [95% CI] = -0.145 [-0.287 to -0.002]; Table 3).

**Table 3:** The difference (95% CIs) in annual changes in total gray matter and total white matter (%) across the three dietary patterns, in men and women (*n* = 1636) ^a, b^

	Total gray matter		Total white matter	
	*β* (95% CI)	*P*-value	*β* (95% CI)	*P*-value
Men (*n* =815)				
Model 1 ^c^				
Estimate (SE) of Intercept	0.141 (0.457)		-0.931 (0.402)	
Vegetable-Fruit-Dairy diet	-0.112 (-0.290 to 0.067)	0.221	0.005 (-0.152 to 0.162)	0.953
Traditional Japanese diet	-0.098 (-0.264 to 0.068)	0.246	0.125 (-0.021 to 0.271)	0.094
Model 2 ^d^				
Estimate (SE) of Intercept	0.207 (0.540)		-0.490 (0.476)	
Vegetable-Fruit-Dairy diet	-0.092 (-0.272 to 0.088)	0.315	-0.023 (-0.181 to 0.136)	0.779
Traditional Japanese diet	-0.088 (-0.254 to 0.079)	0.302	0.103 (-0.044 to 0.249)	0.170
Women (*n* =821)				
Model 1 ^c^				
Estimate (SE) of Intercept	-0.458 (0.551)		-0.416 (0.460)	
Grain-Vegetable-Fruit diet	-0.026 (-0.199 to 0.148)	0.773	-0.093 (-0.238 to 0.052)	0.210
Traditional Japanese diet	-0.149 (-0.291 to -0.007)	0.040	0.070 (-0.048 to 0.188)	0.247
Model 2 ^d^				
Estimate (SE) of Intercept	-0.534 (0.601)		-0.224 (0.500)	
Grain-Vegetable-Fruit diet	-0.024 (-0.198 to 0.150)	0.790	-0.090 (-0.235 to 0.055)	0.223
Tradistional Japanese diet	-0.145 (-0.287 to -0.002)	0.047	0.070 (-0.048 to 0.189)	0.246

^a^ $$\text{Annual changes in region of interest (ROI)}\, (\%)=\frac{\text{relative volume of ROI}_\text{at baseline}-\text{relative volume of ROI}_\text{at follow-up}}{\text{relative volume of ROI}_\text{at baseline}}\div\frac{\text{follow-up time (days)}}{365.25}\times100\%$$

^b^Analyzed by the general linear model. participants in the Western diet were used as the reference group

^c^Adjusted for baseline information on age (years; continuous), APOE genotype (APOE-ε4 carriers: 2/4, 3/4, 4/4, or APOE-ε4 noncarriers: 2/2, 2/3, 3/3), education level (≤ 9, 10 − 12, or ≥ 13 years), smoking status (never, former, or current), total physical activity (METs-h/day; continuous), and energy intake (kcal/day; continuous)

^d^Adjusted for Model 1 plus baseline information on BMI (kg/m^2^; continuous), depressive symptoms (CES-D score; ≤15 or ≥ 16), and medical history (i.e., stroke, hypertension, heart disease, dyslipidemia, and diabetes; dichotomous yes or no responses, for each)

When examining major brain lobes, individuals who adhered to the Traditional Japanese diet had less atrophy in the parietal lobe than those who adhered to the Western diet. Similarly, these individuals also showed a smaller point estimate of atrophy in the frontal lobe; however, the result was only marginally, not statistically, significant (Table 4).

**Table 4:** The difference (95% CIs) in annual changes in brain lobes (%) across the three dietary patterns, in women (*n* = 821) ^a, b, c^

	Intercept	Grain-Vegetable-Fruit diet		Traditional Japanese diet	
	Estimate (SE)	*β* (95% CI)	*P*-value	*β* (95% CI)	*P*-value
Frontal lobe	-0.928 (0.885)	-0.011 (-0.267 to 0.246)	0.935	-0.204 (-0.414 to 0.006)	0.057
Parietal lobe	-1.056 (0.861)	-0.053 (-0.303 to 0.196)	0.676	-0.214 (-0.418 to -0.010)	0.041
Occipital lobe	-0.752 (0.834)	0.022 (-0.220 to 0.263)	0.862	-0.120 (-0.317 to 0.078)	0.236
Temporal lobe	-0.573 (0.753)	-0.067 (-0.285 to 0.151)	0.546	-0.137 (-0.316 to 0.041)	0.133
Insular lobe	-0.624 (0.730)	0.151 (-0.061 to 0.362)	0.163	-0.090 (-0.263 to 0.083)	0.307

^a^ $$\text{Annual changes in region of interest (ROI)}\, (\%)=\frac{\text{relative volume of ROI}_\text{at baseline}-\text{relative volume of ROI}_\text{at follow-up}}{\text{relative volume of ROI}_\text{at baseline}}\div\frac{\text{follow-up time (days)}}{365.25}\times100\%$$

^b^Analyzed by the general linear model. participants in the Western diet were used as the reference group

^c^Adjusted for baseline information on age (years; continuous), APOE genotype (APOE-ε4 carriers: 2/4, 3/4, 4/4, or APOE-ε4 noncarriers: 2/2, 2/3, 3/3), education level (≤ 9, 10 − 12, or ≥ 13 years), smoking status (never, former, or current), total physical activity (METs-h/day; continuous), energy intake (kcal/day; continuous), BMI (kg/m^2^; continuous), depressive symptoms (CES-D score; ≤15 or ≥ 16), and medical history (i.e., stroke, hypertension, heart disease, dyslipidemia, and diabetes; dichotomous yes or no responses, for each)

## Discussion

This longitudinal study that was conducted in a Japanese middle-aged and older community-dwelling population revealed that women who adhered to the Traditional Japanese diet exhibited less brain TGM volume atrophy annually than women who adhered to the Western diet. Furthermore, compared with women who adhered to the Western diet, those who followed the Traditional Japanese diet showed less atrophy in the parietal lobe. However, no association was observed between dietary patterns and brain atrophy in men. To the best of our knowledge, this is the first longitudinal study to explore the association of dietary patterns and brain atrophy in middle-aged and older Japanese populations.

Our study found that, annually, women who adhered to the Traditional Japanese diet had 0.145% less TGM atrophy than women who followed the Western diet. As the average annual TGM atrophy in middle-aged and older women was 0.294%, this suggests that adhering to a Traditional Japanese diet could lead to a 49.3% reduction (0.145/0.294) in TGM atrophy at the population level each year.

To date, only two cross-sectional studies have investigated the relationship between dietary patterns and brain volume in the Japanese population, and the findings of the present study partially confirm the previous findings. One study indicated that adherence to the Traditional Japanese diet (characterized by high consumption of rice, miso, fish and shellfish, green and yellow vegetables, seaweed, pickles, green tea, soybeans and soybean-derived foods, fruits, and mushrooms, but low consumption of beef, pork, and coffee) was inversely associated with WM hyperintensity and cortical atrophy [32]. The Traditional Japanese diet in the present study contained foods that were rich in polyphenols, unsaturated fatty acids, dietary fiber, vitamins, amino acids, and phytochemicals. Thus, our findings may be partly attributable to the antioxidant, anti-inflammatory, and nerve growth-promoting effects of these components [33–37]. Another study involving 171 healthy Japanese individuals reported that those with a high intake of milk and yogurt had a higher normalized GM volume, whereas those who adhered to an “alcohol and animal foods dietary pattern” (with high consumption of alcohol and animal-based foods) had a lower normalized GM volume [38]. However, the Vegetable-Fruit-Dairy diet consumed by men in our present study did not support this finding. Differences in sample size, participants’ age range, dietary assessment method, and statistical analysis approach may contribute to these disparities.

The sex differences observed in dietary patterns and brain atrophy in this study may be attributable to several factors. First, there are sex differences in the role of nutrients and biochemicals that may influence brain integrity. For instance, higher dietary magnesium intake (from fish, shellfish, cereals, legumes, vegetables, fruits, mushrooms, and potatoes) has been associated with larger brain volumes and reduced WM lesions, particularly in women [39]. Additionally, increased whole-grain and legume consumption may increase phytoestrogen intake, potentially reducing the risk of chronic diseases associated with cognitive decline in women [40]. Second, the impact of energy disparities between dietary patterns on the effect of diet on brain atrophy may vary according to sex. An animal experiment reported that a high-fat diet led to the disappearance of differences in the levels of proliferative and neural progenitor cells, which were originally higher in female mice than in male mice [41]. Another study in a female mouse model of Alzheimer’s disease and dementia demonstrated a broader range of metabolic, cognitive, and neuropathological consequences of a high-fat diet than that in male mice [42]. Despite the limited research evidence from humans, the above-described hypotheses align with our findings wherein, compared to the high-fat, high-calorie Western diet, the Traditional Japanese diet exhibited a protective effect against brain atrophy, specifically in women. Third, men’s lifestyle habits may be less favorable than those of women, and thereby potentially offset the brain-protective benefits of dietary patterns. Among our participants, the proportion of current smokers among men at baseline was significantly higher than that among women (21% vs. 4.6%). Previous studies have associated smoking with TGM atrophy in men [43], and our analysis revealed that male smokers had a significantly higher annual average TGM atrophy rate than non-smokers (data not shown). Fifth, although both males and females adhere to the Traditional Japanese diet, there were sex differences in the specific food types consumed. For example, men consumed more noodles (refined carbs) than women and tended to prefer pairing their meals with Japanese sake. Finally, the general reluctance of Japanese men to cook might contribute to the reduced validity of their dietary records compared to that of women, potentially resulting in the misclassification of dietary patterns during data analysis. Ultimately, these factors can impede the ability to ascertain true associations.

After further analysis of the association between dietary patterns and brain-lobe atrophy in women, we observed that women who adhered to the Traditional Japanese diet exhibited less atrophy in their parietal lobe than those who followed the Western diet. A higher MeDi score was associated with greater cortical thickness in the frontal, parietal, and occipital lobes, and average lobar thickness [6], which partially aligns with our findings. However, the current evidence is insufficient to establish a direct link between specific dietary patterns and the integrity of specific brain regions. One possible reason for the lack of associations between dietary patterns and lobes atrophy may be due to the limited statistical power resulting from the relatively small amount of atrophy during the relatively short follow-up period.

 Despite its significant strengths, this study had several limitations that warrant consideration. The dietary survey was conducted only at baseline, precluding the examination of potential dietary changes during the follow-up period and their impact on the results. In addition, using 3-day dietary record for dietary assessment may introduce bias. It is possible that participants opted for healthier food choices than usual during the 3-day dietary record. However, we acknowledge that this reporting bias may have disproportionately affected those who typically follow unhealthy diets, potentially leading to participants with unhealthy dietary habits being categorized into the diet-healthy group. Consequently, this could result in an underestimation of the beneficial effects of the Traditional Japanese diet. The relatively short follow-up time and limited extent of brain atrophy restricted our ability to thoroughly investigate the effects of dietary patterns on regional brain atrophy. Furthermore, despite adjusting for numerous potential confounding factors, the influence of residual confounding factors cannot be excluded entirely. Additionally, we excluded 666 individuals before the analysis. These individuals were older, less educated, more likely to be current smokers and depressed, and had a higher prevalence of medical histories (Additional file Table 14), suggesting that our findings may be limited to relatively healthy community-dwellers. Finally, as an observational study, this research could not establish a causal relationship between the inhibitory effects of dietary patterns on brain atrophy or provide an in-depth exploration of the mechanisms underlying the effects of different dietary patterns. Future observational studies with longitudinal dietary assessments and longer follow-up durations, or randomized controlled trials, are warranted to gain a more thorough understanding of the relationship between dietary patterns and brain health.

## Conclusion

This longitudinal study found that adherence to the Traditional Japanese diet was associated with less brain TGM atrophy in women but not in men. Further studies with longer follow-up periods are warranted to confirm these findings.

### Supplementary Information

**Supplementary Material 1.**

## Data Availability

The datasets analyzed in the current study are not publicly available for privacy reasons but are available from the corresponding author upon reasonable request.

## Abbreviations

APOE: Apoprotein E
BMI: Body mass index
CES-D: Epidemiologic Studies Depression
HCPC: Hierarchical clustering on principal components
MeDi: Mediterranean diet
MFA: Multiple factor analysis
MMSE: Mini-Mental State Examination
MRI: Magnetic resonance imaging
NILS-LSA: National Institute for Longevity Sciences-Longitudinal Study of Aging
PCA: Principal component analysis
PCR: Polymerase chain reaction
TGM: Total gray matter
TWM: Total white matter
WM: White matter
