# Supplementary material for: Associations of dietary patterns and longitudinal brain-volume change in Japanese community-dwelling adults: results from the national institute for longevity sciences-longitudinal study of aging
Source: Nutr J. 2024 Mar 12;23:34. doi: 10.1186/s12937-024-00935-3 (PMC10929119; doi:10.1186/s12937-024-00935-3)
Supplement: Supplementary file 1 — Supplementary Material 1. [file 12937_2024_935_MOESM1_ESM.docx]

| **Table 1**. Dimensions that describe most of the dietary patterns (men; n = 815) ^a^ | | | | |
| --- | --- | --- | --- | --- |
|  |  | Mean in category | Overall mean | *P*-value |
| Western diet | |  |  |  |
|  | Dim.2 | -0.935 | -3.7E-16 | < 0.001 |
|  | Dim.1 | -1.240 | 1.1E-16 | < 0.001 |
|  |  |  |  |  |
| Vegetable-Fruit-Dairy diet | |  |  |  |
|  | Dim.2 | 1.767 | -3.7E-16 | < 0.001 |
|  | Dim.5 | 0.197 | -0.6E-16 | 0.016 |
|  | Dim.4 | -0.225 | -1.4E-16 | 0.007 |
|  | Dim.1 | -0.578 | 1.1E-16 | < 0.001 |
|  |  |  |  |  |
| Traditional Japanese diet | |  |  |  |
|  | Dim.1 | 1.684 | 1.1E-16 | < 0.001 |
|  | Dim.2 | -0.248 | -3.7E-16 | < 0.001 |
| a | A significance threshold of 0.05 was used to select dimensions that characterized the dietary patterns. | | | |

| **Table 2**. The basic information of each food group to the dimensions that are the most associated with dietary patterns (men; n =815) | | | | | | | | | | |
| --- | --- | --- | --- | --- | --- | --- | --- | --- | --- | --- |
| Food groups | | Coordinates | | | |  | Contributions ^a^ | | | |
|  |  | Dim.1 | Dim.2 | Dim.4 | Dim.5 |  | Dim.1 | Dim.2 | Dim.4 | Dim.5 |
| Cereals | | 0.236 | 0.305 | 0.286 | 0.213 |  | 8.342 | 13.169 | 14.825 | 11.524 |
| Fish & shellfish | | 0.173 | 0.193 | 0.120 | 0.133 |  | 6.133 | 8.363 | 6.224 | 7.185 |
| Red meat | | 0.134 | 0.215 | 0.074 | 0.100 |  | 4.736 | 9.293 | 3.852 | 5.434 |
| White meat | | 0.008 | 0.052 | 0.083 | 0.008 |  | 0.299 | 2.244 | 4.308 | 0.436 |
| Eggs | | 0.030 | 0.024 | 0.027 | 0.070 |  | 1.063 | 1.053 | 1.399 | 3.787 |
| Legumes | | 0.189 | 0.044 | 0.103 | 0.090 |  | 6.695 | 1.882 | 5.329 | 4.866 |
| Vegetable | | 0.352 | 0.332 | 0.140 | 0.087 |  | 12.460 | 14.340 | 7.271 | 4.688 |
| Pickles | | 0.079 | 0.045 | 0.066 | 0.135 |  | 2.784 | 1.931 | 3.420 | 7.303 |
| Tubers | | 0.301 | 0.090 | 0.110 | 0.098 |  | 10.646 | 3.909 | 5.680 | 5.302 |
| Mushroom | | 0.111 | 0.030 | 0.026 | 0.014 |  | 3.908 | 1.314 | 1.364 | 0.778 |
| Seaweeds | | 0.069 | 0.036 | 0.038 | 0.007 |  | 2.433 | 1.570 | 1.974 | 0.374 |
| Fruits | | 0.264 | 0.270 | 0.136 | 0.106 |  | 9.322 | 11.652 | 7.030 | 5.728 |
| Dairy | | 0.026 | 0.130 | 0.162 | 0.114 |  | 0.935 | 5.616 | 8.392 | 6.179 |
| Vegetable oils | | 0.160 | 0.230 | 0.115 | 0.041 |  | 5.664 | 9.953 | 5.961 | 2.229 |
| Seasonings | | 0.288 | 0.156 | 0.166 | 0.293 |  | 10.199 | 6.762 | 8.624 | 15.854 |
| Sweets, sugars & sweeteners | | 0.021 | 0.020 | 0.070 | 0.116 |  | 0.737 | 0.870 | 3.643 | 6.301 |
| Green tea | | 0.089 | 0.017 | 0.026 | 0.094 |  | 3.131 | 0.750 | 1.372 | 5.091 |
| Other tea | | 0.091 | 0.014 | 0.007 | 0.032 |  | 3.215 | 0.623 | 0.376 | 1.721 |
| Coffee | | 0.086 | 0.059 | 0.047 | 0.011 |  | 3.050 | 2.561 | 2.424 | 0.606 |
| Soft drinks | | 0.055 | 0.011 | 0.040 | 0.015 |  | 1.944 | 0.487 | 2.059 | 0.832 |
| Alcohol | | 0.065 | 0.038 | 0.086 | 0.070 |  | 2.303 | 1.656 | 4.472 | 3.780 |
| a | Red values indicate the food groups which contributed more than the expected average contribution (1/21×100%) of all food groups. | | | | | | | | | |

| **Table 3**. The basic information of the top 23 (10%) food items to the dimensions that are most associated with dietary patterns (men; n =815) | | | | | | | | | | |
| --- | --- | --- | --- | --- | --- | --- | --- | --- | --- | --- |
| Food items | | Coordinates | | | |  | Contributions ^a^ | | | |
|  |  | Dim.1 | Dim.2 | Dim.4 | Dim.5 |  | Dim.1 | Dim.2 | Dim.4 | Dim.5 |
| Rice, short grain, paddy rice, nonglutinous rice, well-milled, “meshi” (cooked rice) | | 0.171 | -0.208 | -0.272 | -0.176 |  | 0.696 | 1.248 | 2.565 | 1.118 |
| Common wheat, soft flour, first grade | | -0.112 | -0.247 | -0.029 | 0.138 |  | 0.300 | 1.763 | 0.030 | 0.694 |
| Common wheat, bread, white | | -0.210 | 0.195 | 0.417 | 0.184 |  | 1.046 | 1.099 | 6.041 | 1.232 |
| Common wheat, “Somen and Hiyamugi” (thin wheat noodles), dried noodles, boiled | | -0.203 | 0.271 | -0.274 | -0.044 |  | 0.980 | 2.125 | 2.604 | 0.069 |
| Common wheat, yellow alkaline noodles, boiled | | -0.062 | -0.165 | 0.051 | -0.287 |  | 0.091 | 0.793 | 0.091 | 2.980 |
| Common wheat, yellow alkaline noodles, steamed noodles | | -0.085 | -0.221 | 0.067 | 0.084 |  | 0.173 | 1.410 | 0.154 | 0.257 |
| Fish, Pacific saury, with integument, baked | | 0.097 | 0.221 | -0.047 | 0.031 |  | 0.246 | 1.563 | 0.086 | 0.038 |
| Fish, eel, “Kabayaki” (seasoned and baked fillet) | | 0.014 | 0.244 | -0.091 | 0.130 |  | 0.005 | 1.907 | 0.314 | 0.679 |
| Crustacean, giant tiger prawn, cultured, raw | | -0.094 | -0.156 | -0.131 | 0.207 |  | 0.230 | 0.782 | 0.654 | 1.717 |
| Mollusks, Japanese common squid*, raw [*Syn. short-finned squid] | | 0.094 | -0.092 | -0.105 | 0.219 |  | 0.229 | 0.268 | 0.420 | 1.918 |
| Surimi products, “Satsuma-age” (fried surimi) | | 0.234 | -0.196 | -0.096 | 0.065 |  | 1.436 | 1.229 | 0.350 | 0.168 |
| Beef, ground meat, raw | | -0.170 | -0.235 | 0.066 | 0.045 |  | 0.786 | 1.822 | 0.172 | 0.083 |
| Pork, large type breed, picnic shoulder, lean and fat, raw | | -0.092 | -0.196 | 0.038 | 0.094 |  | 0.231 | 1.269 | 0.056 | 0.366 |
| Pork, ground meat, raw | | -0.161 | -0.259 | 0.180 | 0.023 |  | 0.704 | 2.221 | 1.280 | 0.023 |
| Pork, sausage, Vienna | | -0.153 | -0.226 | 0.053 | 0.082 |  | 0.638 | 1.699 | 0.111 | 0.277 |
| Chicken, broiler, breast, meat with skin, raw | | -0.094 | -0.058 | 0.145 | -0.071 |  | 0.289 | 0.134 | 1.011 | 0.251 |
| Chicken, broiler, thigh, meat with skin, boiled | | -0.004 | -0.203 | -0.239 | -0.044 |  | 0.000 | 1.649 | 2.744 | 0.098 |
| Eggs, hen, whole, raw | | 0.091 | -0.005 | 0.125 | -0.239 |  | 0.255 | 0.001 | 0.700 | 2.687 |
| Soybeans, mature seeds, whole, domestic, yellow seed coats, boiled | | 0.205 | 0.078 | 0.054 | 0.015 |  | 1.135 | 0.199 | 0.116 | 0.009 |
| Soybeans, tofu, soft tofu | | -0.027 | 0.028 | -0.177 | 0.019 |  | 0.019 | 0.025 | 1.238 | 0.015 |
| Soybeans, tofu, “Nama-age” (fried slices of drained tofu) | | 0.160 | 0.080 | -0.181 | 0.062 |  | 0.692 | 0.209 | 1.304 | 0.159 |
| Soybeans, tofu, “Abura-age” (fried thin slices of pressed tofu), uncooked | | 0.249 | 0.037 | -0.135 | -0.107 |  | 1.675 | 0.045 | 0.727 | 0.473 |
| Soybeans, natto, “Itohiki-natto” (fermented whole soybean) | | 0.212 | 0.084 | 0.157 | -0.245 |  | 1.220 | 0.236 | 0.980 | 2.490 |
| Carrot, regular (European type), root without skin, boiled | | 0.289 | -0.105 | 0.049 | -0.060 |  | 1.217 | 0.195 | 0.051 | 0.080 |
| Spinach, leaves, all season, boiled | | 0.320 | -0.104 | 0.078 | 0.072 |  | 1.493 | 0.191 | 0.129 | 0.117 |
| Japanese radishes, Daikon, root without skin, boiled | | 0.358 | -0.237 | -0.059 | 0.040 |  | 1.866 | 0.999 | 0.075 | 0.035 |
| Onions, bulb, boiled | | -0.020 | -0.058 | 0.031 | -0.238 |  | 0.006 | 0.059 | 0.021 | 1.264 |
| Eggplant*, Japanese type, fruit, boiled [*Syn. Aubergine] | | -0.140 | 0.349 | -0.152 | -0.097 |  | 0.285 | 2.166 | 0.491 | 0.210 |
| Japanese radishes, Daikon, root, pickles, “Takuan-zuke” (pickled with rice bran and salt), made of sun-dried Daikon | | 0.130 | -0.042 | -0.182 | 0.205 |  | 0.521 | 0.066 | 1.499 | 1.987 |
| Chinese cabbage, head, pickles, salted pickles | | 0.232 | -0.074 | 0.027 | -0.004 |  | 1.668 | 0.205 | 0.032 | 0.001 |
| Chinese cabbage, head, pickles, Kimchi | | 0.009 | -0.081 | 0.140 | -0.313 |  | 0.002 | 0.245 | 0.886 | 4.622 |
| Sweet potato, tuberous root, without skin, raw | | 0.147 | -0.002 | 0.180 | 0.020 |  | 0.682 | 0.000 | 1.493 | 0.018 |
| Taro, “Satoimo”, corm, boiled | | 0.446 | -0.131 | -0.039 | 0.201 |  | 6.247 | 0.655 | 0.071 | 1.947 |
| Potatoes, tuber, boiled | | -0.122 | -0.109 | 0.095 | -0.190 |  | 0.467 | 0.458 | 0.413 | 1.726 |
| Konjac, block, made from fine powder | | 0.260 | -0.178 | -0.160 | 0.070 |  | 2.118 | 1.212 | 1.183 | 0.237 |
| Mushrooms, winter mushrooms*, boiled [*Syn. Enokitake, Enoki] | | 0.223 | -0.098 | 0.160 | 0.134 |  | 1.414 | 0.331 | 1.069 | 0.773 |
| Figs, raw | | 0.011 | 0.254 | -0.171 | 0.012 |  | 0.003 | 1.745 | 0.946 | 0.005 |
| Japanese persimmons*, nonastringent, raw [*Syn. Kaki] | | 0.230 | 0.000 | 0.023 | -0.003 |  | 1.170 | 0.000 | 0.018 | 0.000 |
| Kiwifruit, green flesh type, raw | | 0.002 | 0.116 | 0.145 | 0.195 |  | 0.000 | 0.363 | 0.680 | 1.297 |
| Watermelon, red flesh type, raw | | -0.113 | 0.254 | -0.164 | -0.018 |  | 0.281 | 1.746 | 0.872 | 0.011 |
| Pears, sand pears*, raw [*Syn. Nashi pears] | | -0.067 | 0.224 | -0.117 | 0.044 |  | 0.099 | 1.361 | 0.447 | 0.066 |
| Bananas, raw | | 0.030 | 0.182 | 0.192 | 0.151 |  | 0.019 | 0.904 | 1.201 | 0.777 |
| Grapes, raw | | -0.131 | 0.328 | -0.033 | 0.120 |  | 0.379 | 2.925 | 0.035 | 0.491 |
| Apples, without skin, raw | | 0.400 | -0.011 | 0.071 | 0.070 |  | 3.545 | 0.003 | 0.163 | 0.168 |
| Satsuma mandarins, juice sacs, normal ripening type, raw | | 0.256 | -0.154 | 0.013 | 0.241 |  | 1.460 | 0.643 | 0.006 | 1.974 |
| Liquid milk, whole milk | | 0.065 | 0.115 | 0.200 | 0.243 |  | 0.115 | 0.436 | 1.585 | 2.443 |
| Liquid milk, containing recombined milk, low fat | | 0.040 | 0.051 | 0.169 | -0.144 |  | 0.043 | 0.088 | 1.138 | 0.856 |
| Yogurt, whole milk, unsweetened | | 0.051 | 0.259 | 0.242 | 0.151 |  | 0.071 | 2.226 | 2.332 | 0.946 |
| Cheeses, processed | | -0.048 | 0.083 | 0.263 | 0.114 |  | 0.062 | 0.231 | 2.739 | 0.535 |
| Vegetable oil, blend | | -0.308 | -0.481 | -0.104 | 0.144 |  | 2.889 | 8.640 | 0.481 | 0.970 |
| Fat spread | | -0.216 | 0.068 | 0.347 | 0.148 |  | 1.430 | 0.173 | 5.375 | 1.027 |
| Dressing, mayonnaise, egg yolk type | | -0.210 | -0.175 | 0.049 | -0.070 |  | 1.345 | 1.140 | 0.106 | 0.232 |
| Compound alcoholic beverage, “Mirin” (sweet liquor made from rice, rice koji and Shochu or distilled alcohol), regular | | 0.300 | -0.008 | -0.136 | 0.003 |  | 1.973 | 0.002 | 0.588 | 0.000 |
| Japanese Worcester sauce, common type | | -0.120 | -0.336 | 0.109 | 0.118 |  | 0.314 | 3.010 | 0.379 | 0.468 |
| Soy sauce, “Koikuchi-shoyu” (common soy sauce) | | 0.423 | 0.017 | -0.165 | 0.045 |  | 3.910 | 0.008 | 0.870 | 0.067 |
| Soy sauce, “Usukuchi-shoyu” (light color soy sauce) | | 0.132 | -0.093 | 0.129 | -0.417 |  | 0.383 | 0.230 | 0.534 | 5.820 |
| Soup stock, beef and vegetable stock | | -0.002 | -0.162 | 0.115 | -0.403 |  | 0.000 | 0.702 | 0.424 | 5.417 |
| Japanese noodle soup, non-concentrated (soy sauce base) | | -0.091 | 0.045 | -0.191 | 0.024 |  | 0.179 | 0.054 | 1.164 | 0.019 |
| Roux, Japanese curry roux, instant | | -0.084 | -0.058 | 0.067 | -0.215 |  | 0.156 | 0.088 | 0.146 | 1.541 |
| Ponzu sauce, “Mitsukan Ajipon” (soy sauce with citrus juice) | | 0.168 | -0.008 | 0.179 | 0.045 |  | 0.614 | 0.002 | 1.020 | 0.067 |
| Traditional confectionery, “Arare” (glutinous rice cracker) | | -0.069 | -0.030 | 0.126 | 0.198 |  | 0.124 | 0.029 | 0.608 | 1.558 |
| Traditional confectionery, “Shoyu-senbei” (soy sauce flavored rice cracker) | | -0.058 | 0.004 | 0.138 | -0.218 |  | 0.087 | 0.001 | 0.726 | 1.907 |
| Chocolate, milk chocolate | | 0.000 | 0.003 | 0.162 | 0.183 |  | 0.000 | 0.000 | 0.998 | 1.337 |
| Green tea, “Sencha” (common grade tea), infusion | | 0.282 | 0.017 | 0.083 | -0.117 |  | 2.470 | 0.011 | 0.317 | 0.654 |
| Green tea, “Hoji-cha” (roasted tea), infusion | | 0.130 | -0.080 | 0.107 | 0.287 |  | 0.524 | 0.244 | 0.524 | 3.907 |
| Fermented tea, black tea, infusion | | -0.065 | 0.042 | 0.089 | 0.172 |  | 0.135 | 0.068 | 0.367 | 1.432 |
| “Mugi-cha” (roasted barley tea), infusion | | -0.289 | 0.112 | 0.011 | -0.055 |  | 2.652 | 0.484 | 0.005 | 0.147 |
| Coffee, infusion | | -0.219 | -0.097 | 0.026 | 0.103 |  | 1.454 | 0.350 | 0.030 | 0.491 |
| Coffee, instant coffee, granules | | 0.015 | 0.092 | 0.200 | 0.010 |  | 0.007 | 0.314 | 1.780 | 0.005 |
| Coffee, ready-to-drink coffee with milk and sugar, canned | | -0.208 | -0.222 | -0.100 | 0.041 |  | 1.312 | 1.832 | 0.445 | 0.078 |
| Fermented alcoholic beverage, “Sake”, regular | | 0.176 | -0.076 | -0.165 | -0.022 |  | 0.909 | 0.206 | 1.179 | 0.021 |
| Fermented alcoholic beverage, beer, pale | | -0.119 | -0.044 | -0.199 | -0.038 |  | 0.416 | 0.070 | 1.713 | 0.065 |
| Distilled alcoholic beverage, “Shochu”, distilled through a pot still | | 0.013 | -0.107 | -0.010 | -0.203 |  | 0.005 | 0.412 | 0.005 | 1.853 |
| a | Red values indicate the top 23 (10%) food items to each dimension. | | | | | | | | | |

| **Table 4**. Food items that compose most of the Western diet (men) | | | | | | | | | | |
| --- | --- | --- | --- | --- | --- | --- | --- | --- | --- | --- |
|  |  | Mean consumption volume more than overall mean | | *P*-value |  |  |  | Mean consumption volume less than overall mean | | *P*-value |
|  |  | Mean in category (g/day) | Overall mean (g/day) |  |  |  |  | Mean in category (g/day) | Overall mean (g/day) |  |
| No. of participants | | 310 | 815 |  |  |  |  | 310 | 815 |  |
| Food groups | Food items |  |  |  |  | Food groups | Food items |  |  |  |
| cereals | Common wheat, soft flour, first grade | 3.9 | 2.8 | < 0.001 |  | cereals | Common wheat, “Udon” (thick wheat noodles), boiled | 22.9 | 28.1 | 0.011 |
|  | Common wheat, bread, white long roll | 5.1 | 2.9 | < 0.001 |  |  | Common wheat, “Udon” (thick wheat noodles), dried noodles, boiled | 0.3 | 1.7 | 0.019 |
|  | Common wheat, bread, soft rolls | 5.8 | 4.1 | 0.005 |  |  | Common wheat, “Somen and Hiyamugi” (thin wheat noodles), dried noodles, boiled | 7.8 | 11.6 | 0.018 |
|  | Common wheat, yellow alkaline noodles, boiled | 15.6 | 11.6 | 0.002 |  |  | Rice, glutinous rice products, rice cake | 2.1 | 3.7 | 0.021 |
|  | Common wheat, yellow alkaline noodles, steamed noodles | 11.9 | 6.7 | < 0.001 |  |  | Buckwheat, dried noodles, boiled | 2.9 | 5.6 | 0.007 |
|  | Durum wheat, macaroni and spaghetti, dry pasta, boiled | 16.2 | 11.2 | < 0.001 |  |  | Bun with filling, baked bun with red bean paste filling, regular | 0.8 | 2.2 | 0.002 |
|  | Common wheat, Outer steamed wheat “Jiaozi *” dough (*Chinese meat dumpling) | 1.9 | 1.1 | 0.046 |  | fish & shellfish | Fish, eel, “Kabayaki” (seasoned and baked fillet) | 0.2 | 1.2 | < 0.001 |
|  | Rice, non-glutinous rice products, “Onigiri” (rice ball) | 12.4 | 8.9 | 0.008 |  |  | Fish, skipjack tuna*, caught in spring, raw [*Syn. bonito, skipjack] | 0.5 | 1.1 | 0.049 |
| fish & shellfish | Fish, tuna, canned products, flaked light meat in oil | 1.6 | 1.1 | 0.004 |  |  | Fish, salmon and trout, chum salmon, “Shiozake” (salted salmon), raw | 2.7 | 3.9 | 0.007 |
|  | Crustacean, giant tiger prawn, cultured, raw | 3.4 | 2.4 | < 0.001 |  |  | Fish, Pacific saury, with integument, baked | 1.2 | 3.5 | < 0.001 |
| red meat | Beef, dairy fattened steer, flank or short plate, lean and fat, raw | 2.1 | 1.4 | 0.003 |  |  | Mollusks, short-necked clam*, raw [*Syn. baby-neck clam, Manila clam, Japanese littleneck] | 0.8 | 1.6 | 0.001 |
|  | Beef, ground meat, raw | 5.0 | 2.7 | < 0.001 |  |  | Mollusks, Pacific oyster, cultured, boiled | 0.6 | 1.2 | 0.048 |
|  | Pork, large type breed, picnic shoulder, lean and fat, raw | 9.6 | 6.6 | < 0.001 |  | soybean & soybean products | Soybeans, mature seeds, whole, domestic, yellow seed coats, boiled | 0.5 | 1.2 | < 0.001 |
|  | Pork, large type breed, loin, lean and fat, boiled | 5.5 | 4.1 | 0.014 |  |  | Soybeans, “Budo-mame” (beans cooked with sugar and salt) | 0.5 | 1.4 | < 0.001 |
|  | Pork, ground meat, raw | 5.6 | 3.3 | < 0.001 |  |  | Soybeans, tofu, “Momen-tofu” (regular tofu) | 17.1 | 21.6 | 0.003 |
|  | Pork, bacon | 2.4 | 1.9 | 0.014 |  |  | Soybeans, tofu, “Nama-age” (fried slices of drained tofu) | 1.8 | 3.0 | 0.004 |
|  | Pork, sausage, Vienna | 7.5 | 5.3 | < 0.001 |  |  | Soybeans, tofu, “Abura-age” (fried thin slices of pressed tofu), uncooked | 2.1 | 3.3 | < 0.001 |
|  | Pork, roast pork | 2.2 | 1.4 | 0.002 |  |  | Soybeans, natto, “Itohiki-natto” (fermented whole soybean) | 5.4 | 8.0 | < 0.001 |
| white meat | Chicken, broiler, breast, meat with skin, raw | 2.6 | 1.6 | 0.002 |  | vegetable | Kidney beans, “Sayaingen”, immature pods, boiled | 1.2 | 1.8 | 0.042 |
|  | Chicken, broiler, thigh, meat with skin, boiled | 15.7 | 12.8 | 0.005 |  |  | Pumpkin and squash, winter squash*, fruit, boiled [*Syn. Pumpkin] | 3.7 | 7.8 | < 0.001 |
| eggs | Eggs, hen, Tamago-yaki (Rolled omelet), Atsuyaki-tamago (sweet rolled omelet with Katsuo-bushi and kombu dashi) | 2.0 | 1.4 | 0.017 |  |  | Cucumber, fruit, raw | 13.2 | 15.4 | 0.032 |
| soybean & soybean products | Soybeans, tofu, “Juten-tofu” (packaged tofu) | 2.8 | 1.6 | 0.009 |  |  | Edible burdock, root, boiled | 3.1 | 4.2 | 0.003 |
| vegetable | Cabbage, common, head, raw | 13.6 | 11.8 | 0.028 |  |  | Spinach mustard, “Komatsuna”, leaves, boiled | 1.3 | 2.9 | < 0.001 |
|  | Bean sprouts, mung bean sprouts, boiled | 9.8 | 8.2 | 0.020 |  |  | Japanese radishes, Daikon, leaves, boiled | 0.3 | 1.1 | 0.005 |
|  | Vegetable juice, “Ninjin 55” (Japanese carrot-based, salt free) | 16.9 | 10.7 | 0.001 |  |  | Japanese radishes, Daikon, root without skin, raw | 7.2 | 9.9 | 0.001 |
| tubers | Potatoes, tuber, boiled | 26.1 | 22.2 | 0.001 |  |  | Japanese radishes, Daikon, root without skin, boiled | 12.6 | 17.7 | < 0.001 |
| vegetable oils | Vegetable oil, blend | 7.8 | 5.5 | < 0.001 |  |  | Japanese radishes, Daikon, “Kiriboshi-daikon” (cut and dried Daikon root), raw | 1.6 | 3.8 | 0.001 |
|  | Fat spread | 1.7 | 1.3 | 0.009 |  |  | Bamboo shoots, boiled | 1.4 | 2.5 | 0.027 |
|  | Dressing, mayonnaise, egg yolk type | 4.5 | 3.4 | < 0.001 |  |  | Chinese preserving melon, fruit, boiled | 0.3 | 1.8 | 0.002 |
| seasonings | Japanese Worcester sauce, common type | 2.9 | 2.0 | < 0.001 |  |  | Tomatoes, fruit, raw | 15.5 | 23.2 | < 0.001 |
|  | Soup stock, beef and vegetable stock | 3.9 | 2.8 | 0.002 |  |  | Eggplant*, Japanese type, fruit, boiled [*Syn. Aubergine] | 6.5 | 9.3 | 0.001 |
|  | Tomato products, ketchup | 2.2 | 1.6 | < 0.001 |  |  | Bitter melon, fruit, raw | 0.4 | 1.5 | 0.001 |
|  | Roux, Japanese curry roux, instant | 1.7 | 1.2 | < 0.001 |  |  | Carrot, regular (European type), root without skin, boiled | 14.9 | 16.9 | 0.006 |
| other tea | “Mugi-cha” (roasted barley tea), infusion | 128.3 | 99.8 | 0.005 |  |  | Welsh onions, “Ha-negi” (large variety, green), leaves, raw | 4.4 | 5.6 | 0.011 |
| coffee | Coffee, infusion | 163.7 | 121.1 | < 0.001 |  |  | Broccoli, inflorescence, boiled | 4.3 | 5.9 | 0.003 |
|  | Coffee, ready-to-drink coffee with milk and sugar, canned | 40.4 | 20.1 | < 0.001 |  |  | Spinach, leaves, all season, boiled | 7.9 | 10.8 | 0.001 |
| soft drinks | Carbonated beverage, cola | 5.7 | 2.9 | 0.003 |  | pickles | Chinese cabbage, head, pickles, salted pickles | 0.6 | 1.9 | 0.002 |
|  | Carbonated beverage, clear soda | 7.9 | 3.8 | 0.001 |  |  | Mume*, “Umeboshi” (pickled and dried mume), seasoned pickles [*Syn. Japanese apricots] | 0.8 | 1.0 | 0.034 |
|  | Sports drink, “Pocari Sweat” | 29.8 | 19.6 | 0.004 |  | tubers | Konjac, block, made from fine powder | 2.1 | 3.8 | < 0.001 |
| alcohol | Fermented alcoholic beverage, beer, pale | 91.0 | 74.3 | 0.033 |  |  | Sweet potato, tuberous root, without skin, raw | 0.9 | 1.6 | 0.032 |
|  | Fermented alcoholic beverage, “Happoshu” (beer-like beverage with less than 67% malt content) | 100.7 | 81.4 | 0.022 |  |  | Taro, “Satoimo”, corm, boiled | 3.0 | 8.1 | < 0.001 |
|  | Shochu highball, “Takohai Lemon Lime” | 13.1 | 6.5 | 0.001 |  |  | Yam, Chinese yam, “Nagaimo”, tuberous root, raw | 1.4 | 3.1 | 0.001 |
|  |  |  |  |  |  | mushroom | Mushrooms, “Shiitake”, boiled | 1.3 | 2.0 | 0.006 |
|  |  |  |  |  |  |  | Mushrooms, “Shiitake”, dried, boiled | 0.7 | 1.2 | 0.001 |
|  |  |  |  |  |  |  | Mushrooms, beech mushrooms, boiled | 1.9 | 2.5 | 0.015 |
|  |  |  |  |  |  |  | Mushrooms, king oyster mushrooms, raw | 0.4 | 1.0 | 0.002 |
|  |  |  |  |  |  | seaweeds | Algae, “Hijiki”, dried | 1.3 | 2.5 | 0.001 |
|  |  |  |  |  |  |  | Algae, “Mozuku”, salted products, desalted | 0.6 | 1.8 | < 0.001 |
|  |  |  |  |  |  |  | Algae, “Wakame”, cut and dried | 5.5 | 6.8 | 0.018 |
|  |  |  |  |  |  |  | Algae, “Wakame”, blanched and salted products, desalted | 1.5 | 2.7 | < 0.001 |
|  |  |  |  |  |  | fruits | Strawberries, raw | 1.6 | 2.9 | 0.011 |
|  |  |  |  |  |  |  | Figs, raw | 0.1 | 1.6 | 0.006 |
|  |  |  |  |  |  |  | Satsuma mandarins, segments, normal ripening type, raw | 1.5 | 5.6 | 0.028 |
|  |  |  |  |  |  |  | Japanese persimmons*, nonastringent, raw [*Syn. Kaki] | 2.6 | 7.4 | < 0.001 |
|  |  |  |  |  |  |  | Watermelon, red flesh type, raw | 2.8 | 6.1 | 0.033 |
|  |  |  |  |  |  |  | Pears, sand pears*, raw [*Syn. Nashi pears] | 3.3 | 6.1 | 0.011 |
|  |  |  |  |  |  |  | Bananas, raw | 19.9 | 25.6 | 0.001 |
|  |  |  |  |  |  |  | Grapes, raw | 2.0 | 3.9 | 0.015 |
|  |  |  |  |  |  |  | Muskmelon, greenhouse culture, raw | 0.3 | 1.1 | 0.036 |
|  |  |  |  |  |  |  | Apples, without skin, raw | 9.4 | 18.3 | < 0.001 |
|  |  |  |  |  |  | dairy & dairy products | Liquid milk, whole milk | 54.1 | 67.2 | 0.002 |
|  |  |  |  |  |  |  | Yogurt, whole milk, unsweetened | 9.0 | 16.2 | < 0.001 |
|  |  |  |  |  |  | seasonings | Compound alcoholic beverage, “Mirin” (sweet liquor made from rice, rice koji and Shochu or distilled alcohol), regular | 2.3 | 3.1 | < 0.001 |
|  |  |  |  |  |  |  | Soy sauce, “Koikuchi-shoyu” (common soy sauce) | 10.2 | 13.4 | < 0.001 |
|  |  |  |  |  |  |  | Vinegar, grain vinegar | 1.1 | 1.8 | < 0.001 |
|  |  |  |  |  |  |  | Japanese noodle soup, triple-concentrated (soy sauce base) | 3.5 | 4.5 | 0.003 |
|  |  |  |  |  |  |  | Miso, rice-koji miso, red type | 3.1 | 4.1 | < 0.001 |
|  |  |  |  |  |  |  | Miso, soybean-koji miso | 3.0 | 4.1 | < 0.001 |
|  |  |  |  |  |  |  | Ready-made miso (miso-based sauce) | 1.0 | 1.3 | 0.027 |
|  |  |  |  |  |  | green tea | Green tea, “Sencha” (common grade tea), infusion | 235.5 | 311.9 | < 0.001 |
|  |  |  |  |  |  |  | Green tea, “Ban-cha” (coarse grade tea), infusion | 12.0 | 30.5 | 0.003 |
|  |  |  |  |  |  |  | Green tea, “Genmai-cha” (mixture of tea and roasted brown rice), infusion | 3.4 | 15.3 | 0.003 |
|  |  |  |  |  |  | alcohol | Fermented alcoholic beverage, “Sake”, regular | 11.2 | 21.6 | < 0.001 |

| **Table 5**. Food items that compose most of the Vegetable-Fruit-Dairy diet (men) | | | | | | | | | | |
| --- | --- | --- | --- | --- | --- | --- | --- | --- | --- | --- |
|  | | Mean consumption volume more than overall mean | | *P*-value |  |  |  | Mean consumption volume less than overall mean | | *P*-value |
|  |  | Mean in category (g/day) | Overall mean (g/day) |  |  |  |  | Mean in category (g/day) | Overall mean (g/day) |  |
| No. of participants | | 206 | 815 |  |  |  |  | 206 | 815 |  |
| Food groups | Food items |  |  |  |  | Food groups | Food items |  |  |  |
| cereals | Common wheat, bread, white | 36.6 | 27.6 | < 0.001 |  | cereals | Common wheat, soft flour, first grade | 1.6 | 2.8 | < 0.001 |
|  | Common wheat, “Somen and Hiyamugi” (thin wheat noodles), dried noodles, boiled | 30.0 | 11.6 | < 0.001 |  |  | Common wheat, bread, soft rolls | 2.2 | 4.1 | 0.014 |
|  | Rice, short grain, paddy rice, under-milled, “meshi” (cooked rice) | 5.8 | 2.5 | 0.039 |  |  | Common wheat, yellow alkaline noodles, steamed noodles | 2.4 | 6.7 | < 0.001 |
|  | Buckwheat, dried noodles, boiled | 11.7 | 5.6 | < 0.001 |  |  | Rice, short grain, paddy rice, nonglutinous rice, well-milled, “meshi” (cooked rice) | 282.9 | 325.1 | < 0.001 |
|  | Rice, “Sushi Rice” (with vinegar) | 19.8 | 14.0 | 0.002 |  | fish & shellfish | Mollusks, Pacific oyster, cultured, boiled | 0.2 | 1.2 | 0.017 |
| fish & shellfish | Fish, eel, “Kabayaki” (seasoned and baked fillet) | 3.5 | 1.2 | < 0.001 |  |  | Crustacean, giant tiger prawn, cultured, raw | 1.5 | 2.4 | 0.025 |
|  | Fish, salmon and trout, chum salmon, “Shiozake” (salted salmon), raw | 5.5 | 3.9 | 0.006 |  |  | Surimi products, “Mushi-kamaboko” (steamed kamaboko) | 1.0 | 1.7 | 0.007 |
|  | Fish, mackerel, processed products, “Shiosaba” (plain salted fillet) | 3.0 | 2.0 | 0.042 |  |  | Surimi products, “Yaki-chikuwa” (baked tubular kamaboko) | 2.3 | 3.3 | 0.020 |
|  | Fish, Pacific saury, with integument, baked | 7.4 | 3.5 | < 0.001 |  |  | Surimi products, “Satsuma-age” (fried surimi) | 1.7 | 3.7 | < 0.001 |
|  | Mollusks, short-necked clam*, raw [*Syn. baby-neck clam, Manila clam, Japanese littleneck] | 2.5 | 1.6 | 0.007 |  | red meat | Beef, ground meat, raw | 0.7 | 2.7 | < 0.001 |
|  | Mollusks, common octopus, boiled | 2.7 | 1.8 | 0.025 |  |  | Pork, large type breed, picnic shoulder, lean and fat, raw | 3.8 | 6.6 | 0.001 |
| soybean & soybean products | Soybeans, tofu, “Kinugoshi-tofu” (silken tofu) | 13.7 | 8.5 | < 0.001 |  |  | Pork, large type breed, inside ham, without subcutaneous fat, boiled | 2.8 | 4.6 | 0.022 |
|  | Soybeans, tofu, soft tofu | 12.0 | 8.8 | 0.011 |  |  | Pork, ground meat, raw | 1.1 | 3.3 | < 0.001 |
| vegetable | Kidney beans, “Sayaingen”, immature pods, boiled | 3.3 | 1.8 | < 0.001 |  |  | Pork, sausage, Vienna | 3.3 | 5.3 | 0.001 |
|  | Okra, pods, boiled | 3.2 | 1.5 | < 0.001 |  | white meat | Chicken, broiler, thigh, meat with skin, boiled | 8.7 | 12.8 | 0.003 |
|  | Pumpkin and squash, winter squash*, fruit, boiled [*Syn. Pumpkin] | 11.0 | 7.8 | 0.001 |  | vegetable | Cabbage, common, head, boiled | 10.0 | 13.8 | 0.003 |
|  | Cucumber, fruit, raw | 25.3 | 15.4 | < 0.001 |  |  | Japanese radishes, Daikon, leaves, boiled | 0.2 | 1.1 | 0.021 |
|  | Onions, bulb, raw | 3.6 | 2.3 | 0.011 |  |  | Japanese radishes, Daikon, root without skin, boiled | 8.2 | 17.7 | < 0.001 |
|  | Chinese preserving melon, fruit, boiled | 5.6 | 1.8 | < 0.001 |  |  | Japanese radishes, Daikon, “Kiriboshi-daikon” (cut and dried Daikon root), raw | 1.9 | 3.8 | 0.033 |
|  | Corn, sweet corn, immature kernels, boiled | 3.0 | 1.3 | < 0.001 |  |  | Onions, bulb, boiled | 24.9 | 28.4 | 0.043 |
|  | Tomatoes, fruit, raw | 35.6 | 23.2 | < 0.001 |  |  | Carrot, regular (European type), root without skin, boiled | 12.4 | 16.9 | < 0.001 |
|  | Tomatoes, cherry tomatoes, fruit, raw | 6.8 | 4.4 | 0.001 |  |  | Welsh onions, “Nebuka-negi” (large variety, blanching cultivation), leaves, raw | 1.8 | 3.8 | < 0.001 |
|  | Tomatoes, canned products, tomato-based vegetable juice, with salt | 8.9 | 3.5 | 0.001 |  |  | Welsh onions, “Ha-negi” (large variety, green), leaves, raw | 4.3 | 5.6 | 0.035 |
|  | Eggplant*, Japanese type, fruit, boiled [*Syn. Aubergine] | 19.0 | 9.3 | < 0.001 |  |  | Chinese cabbage, head, boiled | 3.7 | 12.9 | < 0.001 |
|  | Bitter melon, fruit, raw | 4.4 | 1.5 | < 0.001 |  |  | Broccoli, inflorescence, boiled | 3.6 | 5.9 | 0.003 |
|  | Sweet peppers, fruit, green, raw | 5.5 | 4.0 | < 0.001 |  |  | Spinach, leaves, all season, boiled | 5.8 | 10.8 | < 0.001 |
|  | Lettuce, head lettuce, crisp type, soil culture, head, raw | 13.1 | 9.6 | < 0.001 |  |  | East Indian lotus root, rhizome, boiled | 1.7 | 2.6 | 0.040 |
| pickles | Cucumber, fruit, pickles, salted pickles | 3.5 | 1.9 | < 0.001 |  | tubers | Konjac, block, made from fine powder | 2.4 | 3.8 | 0.019 |
| tubers | Yam, Chinese yam, “Nagaimo”, tuberous root, raw | 4.9 | 3.1 | 0.004 |  |  | Konjac, noodles | 0.8 | 2.0 | 0.002 |
| mushroom | Mushrooms, “Shiitake”, dried, boiled | 1.6 | 1.2 | 0.008 |  |  | Taro, “Satoimo”, corm, boiled | 3.3 | 8.1 | < 0.001 |
| seaweeds | Algae, “Wakame”, blanched and salted products, desalted | 3.9 | 2.7 | < 0.001 |  | mushroom | Mushrooms, winter mushrooms*, boiled [*Syn. Enokitake, Enoki] | 1.6 | 2.8 | 0.001 |
| fruits | Figs, raw | 5.2 | 1.6 | < 0.001 |  | fruits | Satsuma mandarins, juice sacs, normal ripening type, raw | 1.2 | 8.9 | < 0.001 |
|  | Oranges, Valencia, imported from the U.S.A., juice sacs, raw | 3.5 | 1.8 | 0.001 |  |  | Japanese persimmons*, nonastringent, raw [*Syn. Kaki] | 2.6 | 7.4 | 0.006 |
|  | Kiwifruit, green flesh type, raw | 4.5 | 2.9 | 0.010 |  |  | Apples, without skin, raw | 9.2 | 18.3 | < 0.001 |
|  | Watermelon, red flesh type, raw | 18.7 | 6.1 | < 0.001 |  | vegetable oils | Vegetable oil, blend | 3.5 | 5.5 | < 0.001 |
|  | Pears, sand pears*, raw [*Syn. Nashi pears] | 13.6 | 6.1 | < 0.001 |  |  | Dressing, mayonnaise, egg yolk type | 2.7 | 3.4 | 0.012 |
|  | Bananas, raw | 32.4 | 25.6 | 0.003 |  | seasonings | Compound alcoholic beverage, “Mirin” (sweet liquor made from rice, rice koji and Shochu or distilled alcohol), regular | 2.5 | 3.1 | 0.011 |
|  | Grapes, raw | 11.1 | 3.9 | < 0.001 |  |  | Japanese Worcester sauce, common type | 0.9 | 2.0 | < 0.001 |
|  | Muskmelon, greenhouse culture, raw | 3.5 | 1.1 | < 0.001 |  |  | Soy sauce, “Usukuchi-shoyu” (light color soy sauce) | 0.9 | 1.6 | < 0.001 |
|  | Peaches, raw | 4.0 | 1.2 | < 0.001 |  |  | Soup stock, beef and vegetable stock | 0.8 | 2.8 | < 0.001 |
|  | Apples, straight fruit juice | 2.1 | 1.0 | 0.017 |  |  | Tomato products, ketchup | 1.1 | 1.6 | 0.037 |
| dairy & dairy products | Liquid milk, whole milk | 79.1 | 67.2 | 0.034 |  |  | Roux, Japanese curry roux, instant | 0.7 | 1.2 | 0.024 |
|  | Yogurt, whole milk, unsweetened | 26.7 | 16.2 | < 0.001 |  |  | Ponzu sauce, “Mitsukan Ajipon” (soy sauce with citrus juice) | 1.0 | 1.4 | 0.040 |
|  | Yogurt, skimmed, sweetened | 14.8 | 9.4 | < 0.001 |  | sweets, sugars & sweeteners | Cake and pastry, Danish pastry | 0.8 | 2.0 | 0.037 |
|  | Lactic acid drink, “Meiji Love Ace” (high iron) | 12.2 | 3.5 | < 0.001 |  | coffee | Coffee, ready-to-drink coffee with milk and sugar, canned | 9.9 | 20.1 | 0.010 |
| vegetable oils | Fat spread | 1.8 | 1.3 | 0.005 |  | alcohol | Shochu highball, “Takohai Lemon Lime” | 0.4 | 6.5 | 0.025 |
| seasonings | Vinegar, grain vinegar | 2.5 | 1.8 | 0.002 |  |  |  |  |  |  |
|  | Japanese noodle soup, non-concentrated (soy sauce base) | 6.2 | 4.2 | 0.007 |  |  |  |  |  |  |
|  | Japanese noodle soup, triple-concentrated (soy sauce base) | 6.2 | 4.5 | < 0.001 |  |  |  |  |  |  |
| green tea | Green tea, “Genmai-cha” (mixture of tea and roasted brown rice), infusion | 26.2 | 15.3 | 0.042 |  |  |  |  |  |  |
| other tea | Fermented tea, Oolong tea, infusion | 108.4 | 75.4 | 0.008 |  |  |  |  |  |  |
|  | “Mugi-cha” (roasted barley tea), infusion | 164.3 | 99.8 | < 0.001 |  |  |  |  |  |  |
| coffee | Coffee, instant coffee, granules | 1.5 | 1.2 | 0.016 |  |  |  |  |  |  |
| alcohol | Fermented alcoholic beverage, wine, red | 5.3 | 2.4 | 0.045 |  |  |  |  |  |  |

| **Table 6**. Food items that compose most of the Traditional Japanese diet (men) | | | | | | | | | | |
| --- | --- | --- | --- | --- | --- | --- | --- | --- | --- | --- |
|  | | Mean consumption volume more than overall mean | | *P*-value |  |  |  | Mean consumption volume less than overall mean | | *P*-value |
|  |  | Mean in category (g/day) | Overall mean (g/day) |  |  |  |  | Mean in category (g/day) | Overall mean (g/day) |  |
| No. of participants | | 299 | 815 |  |  |  |  | 299 | 815 |  |
| Food groups | Food items |  |  |  |  | Food groups | Food items |  |  |  |
| cereals | Common wheat, “Udon” (thick wheat noodles), boiled | 36.1 | 28.1 | < 0.001 |  | cereals | Common wheat, bread, white | 20.1 | 27.6 | < 0.001 |
|  | Common wheat, “Udon” (thick wheat noodles), dried noodles, boiled | 3.2 | 1.7 | 0.010 |  |  | Common wheat, bread, white long roll | 1.5 | 2.9 | 0.005 |
|  | Rice, short grain, paddy rice, half-milled, “meshi” (cooked rice) | 4.7 | 1.8 | 0.036 |  |  | Common wheat, “Somen and Hiyamugi” (thin wheat noodles), dried noodles, boiled | 3.0 | 11.6 | < 0.001 |
|  | Rice, short grain, paddy rice, nonglutinous rice, well-milled, “meshi” (cooked rice) | 357.1 | 325.1 | < 0.001 |  |  | Common wheat, yellow alkaline noodles, steamed noodles | 4.4 | 6.7 | 0.006 |
|  | Rice, glutinous rice products, rice cake | 6.6 | 3.7 | < 0.001 |  |  | Durum wheat, macaroni and spaghetti, dry pasta, boiled | 5.4 | 11.2 | < 0.001 |
| fish & shellfish | Fish, horse mackerel, Japanese Jack mackerel＊, “Hirakiboshi” (salted and semi-dried split), baked [*Syn. horse mackerel] | 3.1 | 2.3 | 0.028 |  |  | Rice, non-glutinous rice products, “Onigiri” (rice ball) | 4.1 | 8.9 | < 0.001 |
|  | Fish, yellowtail*, mature, raw [*Syn. five-ray yellowtail] | 4.1 | 3.0 | 0.017 |  |  | Rice, “Sushi Rice” (with vinegar) | 10.1 | 14.0 | 0.008 |
|  | Mollusks, Pacific oyster, cultured, boiled | 2.6 | 1.2 | < 0.001 |  | fish & shellfish | Fish, tuna, canned products, flaked light meat in oil | 0.8 | 1.1 | 0.025 |
|  | Mollusks, Japanese common squid*, baked [*Syn. short-finned squid] | 4.3 | 3.3 | 0.020 |  | red meat | Beef, dairy fattened steer, flank or short plate, lean and fat, raw | 0.8 | 1.4 | 0.034 |
|  | Surimi products, “Mushi-kamaboko” (steamed kamaboko) | 2.5 | 1.7 | < 0.001 |  |  | Beef, ground meat, raw | 1.7 | 2.7 | 0.001 |
|  | Surimi products, “Yaki-chikuwa” (baked tubular kamaboko) | 4.4 | 3.3 | 0.001 |  |  | Pork, large type breed, loin, lean and fat, boiled | 2.3 | 4.1 | 0.002 |
|  | Surimi products, “Satsuma-age” (fried surimi) | 5.9 | 3.7 | < 0.001 |  |  | Pork, ground meat, raw | 2.4 | 3.3 | 0.009 |
| eggs | Eggs, hen, whole, raw | 3.5 | 2.7 | 0.037 |  |  | Pork, sausage, Vienna | 4.3 | 5.3 | 0.032 |
| soybean & soybean products | Soybeans, mature seeds, whole, domestic, yellow seed coats, boiled | 2.2 | 1.2 | < 0.001 |  |  | Pork, roast pork | 0.9 | 1.4 | 0.043 |
|  | Soybeans, “Budo-mame” (beans cooked with sugar and salt) | 2.5 | 1.4 | < 0.001 |  | soybean & soybean products | Soybeans, tofu, “Kinugoshi-tofu” (silken tofu) | 5.8 | 8.5 | 0.009 |
|  | Soybeans, tofu, “Momen-tofu” (regular tofu) | 27.4 | 21.6 | < 0.001 |  | vegetable | Okra, pods, boiled | 0.8 | 1.5 | 0.010 |
|  | Soybeans, tofu, “Nama-age” (fried slices of drained tofu) | 4.0 | 3.0 | 0.033 |  |  | Cucumber, fruit, raw | 10.7 | 15.4 | < 0.001 |
|  | Soybeans, tofu, “Abura-age” (fried thin slices of pressed tofu), uncooked | 4.6 | 3.3 | < 0.001 |  |  | Chinese preserving melon, fruit, boiled | 0.8 | 1.8 | 0.041 |
|  | Soybeans, natto, “Itohiki-natto” (fermented whole soybean) | 11.1 | 8.0 | < 0.001 |  |  | Corn, sweet corn, immature kernels, boiled | 0.3 | 1.3 | 0.002 |
| vegetable | Pumpkin and squash, winter squash*, fruit, boiled [*Syn. Pumpkin] | 9.9 | 7.8 | 0.006 |  |  | Eggplant*, Japanese type, fruit, boiled [*Syn. Aubergine] | 5.6 | 9.3 | < 0.001 |
|  | Edible burdock, root, boiled | 5.5 | 4.2 | < 0.001 |  |  | Bitter melon, fruit, raw | 0.5 | 1.5 | 0.006 |
|  | Spinach mustard, “Komatsuna”, leaves, boiled | 4.6 | 2.9 | < 0.001 |  |  | Lettuce, head lettuce, crisp type, soil culture, head, raw | 7.8 | 9.6 | 0.009 |
|  | Japanese radishes, Daikon, leaves, boiled | 2.4 | 1.1 | < 0.001 |  |  | Vegetable juice, “Ninjin 55” (Japanese carrot-based, salt free) | 6.3 | 10.7 | 0.022 |
|  | Japanese radishes, Daikon, root without skin, raw | 11.9 | 9.9 | 0.016 |  | pickles | Cucumber, fruit, pickles, salted pickles | 1.0 | 1.9 | 0.013 |
|  | Japanese radishes, Daikon, root without skin, boiled | 29.6 | 17.7 | < 0.001 |  | fruits | Watermelon, red flesh type, raw | 0.8 | 6.1 | 0.001 |
|  | Japanese radishes, Daikon, “Kiriboshi-daikon” (cut and dried Daikon root), raw | 7.3 | 3.8 | < 0.001 |  |  | Pears, sand pears*, raw [*Syn. Nashi pears] | 3.9 | 6.1 | 0.049 |
|  | Bamboo shoots, boiled | 4.0 | 2.5 | 0.004 |  |  | Grapes, raw | 0.8 | 3.9 | < 0.001 |
|  | Green bok choy, leaves, boiled | 2.0 | 1.4 | 0.018 |  |  | Muskmelon, open culture, green flesh type, raw | 0.4 | 2.1 | 0.004 |
|  | Carrot, regular (European type), root without skin, boiled | 22.1 | 16.9 | < 0.001 |  |  | Peaches, raw | 0.0 | 1.2 | 0.004 |
|  | Welsh onions, “Nebuka-negi” (large variety, blanching cultivation), leaves, raw | 5.4 | 3.8 | < 0.001 |  | dairy & dairy products | Yogurt, skimmed, sweetened | 6.1 | 9.4 | 0.003 |
|  | Welsh onions, “Ha-negi” (large variety, green), leaves, raw | 7.8 | 5.6 | < 0.001 |  | vegetable oils | Vegetable oil, blend | 4.5 | 5.5 | < 0.001 |
|  | Chinese cabbage, head, boiled | 21.4 | 12.9 | < 0.001 |  |  | Fat spread | 0.7 | 1.3 | < 0.001 |
|  | Broccoli, inflorescence, boiled | 9.3 | 5.9 | < 0.001 |  |  | Dressing, mayonnaise, egg yolk type | 2.6 | 3.4 | < 0.001 |
|  | Spinach, leaves, all season, boiled | 17.1 | 10.8 | < 0.001 |  | seasonings | Japanese noodle soup, non-concentrated (soy sauce base) | 2.6 | 4.2 | 0.003 |
|  | East Indian lotus root, rhizome, boiled | 3.7 | 2.6 | < 0.001 |  |  | Tomato products, ketchup | 1.3 | 1.6 | 0.039 |
| pickles | Japanese radishes, Daikon, root, pickles, “Takuan-zuke” (pickled with rice bran and salt), made of salted Daikon | 2.5 | 1.8 | 0.026 |  | other tea | Fermented tea, Oolong tea, infusion | 51.3 | 75.4 | 0.012 |
|  | Japanese radishes, Daikon, root, pickles, “Takuan-zuke” (pickled with rice bran and salt), made of sun-dried Daikon | 2.4 | 1.5 | 0.001 |  |  | “Mugi-cha” (roasted barley tea), infusion | 25.9 | 99.8 | < 0.001 |
|  | Chinese cabbage, head, pickles, salted pickles | 3.8 | 1.9 | < 0.001 |  | coffee | Coffee, infusion | 78.8 | 121.1 | < 0.001 |
| tubers | Konjac, block, made from fine powder | 6.5 | 3.8 | < 0.001 |  |  | Coffee, ready-to-drink coffee with milk and sugar, canned | 6.2 | 20.1 | < 0.001 |
|  | Konjac, noodles | 3.1 | 2.0 | < 0.001 |  | soft drinks | Carbonated beverage, fruit flavored and colored drink | 0.0 | 2.9 | 0.043 |
|  | Sweet potato, tuberous root, without skin, raw | 3.0 | 1.6 | < 0.001 |  |  | Carbonated beverage, cola | 0.1 | 2.9 | 0.004 |
|  | Taro, “Satoimo”, corm, boiled | 16.8 | 8.1 | < 0.001 |  |  | Carbonated beverage, clear soda | 0.3 | 3.8 | 0.008 |
| mushroom | Mushrooms, winter mushrooms*, boiled [*Syn. Enokitake, Enoki] | 4.2 | 2.8 | < 0.001 |  |  | Sports drink, “Pocari Sweat” | 11.2 | 19.6 | 0.025 |
|  | Mushrooms, “Shiitake”, boiled | 2.9 | 2.0 | 0.001 |  | alcohol | Fermented alcoholic beverage, beer, pale | 53.2 | 74.3 | 0.009 |
|  | Mushrooms, beech mushrooms, boiled | 3.5 | 2.5 | < 0.001 |  |  | Fermented alcoholic beverage, “Happoshu” (beer-like beverage with less than 67% malt content) | 50.3 | 81.4 | < 0.001 |
| seaweeds | Algae, “Hijiki”, dried | 3.9 | 2.5 | < 0.001 |  |  |  |  |  |  |
|  | Algae, “Mozuku”, salted products, desalted | 2.6 | 1.8 | 0.004 |  |  |  |  |  |  |
|  | Algae, “Wakame”, cut and dried | 8.0 | 6.8 | 0.032 |  |  |  |  |  |  |
| fruits | Strawberries, raw | 4.7 | 2.9 | < 0.001 |  |  |  |  |  |  |
|  | Citrus, “Iyo”, juice sacs | 2.3 | 1.1 | 0.009 |  |  |  |  |  |  |
|  | Satsuma mandarins, segments, early ripening type, raw | 3.2 | 1.8 | 0.015 |  |  |  |  |  |  |
|  | Satsuma mandarins, segments, normal ripening type, raw | 12.6 | 5.6 | < 0.001 |  |  |  |  |  |  |
|  | Satsuma mandarins, juice sacs, normal ripening type, raw | 17.3 | 8.9 | < 0.001 |  |  |  |  |  |  |
|  | Japanese persimmons*, nonastringent, raw [*Syn. Kaki] | 15.7 | 7.4 | < 0.001 |  |  |  |  |  |  |
|  | Grapefruit, white flesh type, juice sacs, raw | 2.3 | 1.4 | 0.047 |  |  |  |  |  |  |
|  | Citrus, “Hassaku”, juice sacs, raw | 3.4 | 1.7 | 0.001 |  |  |  |  |  |  |
|  | Apples, without skin, raw | 33.7 | 18.3 | < 0.001 |  |  |  |  |  |  |
| seasonings | Compound alcoholic beverage, “Mirin” (sweet liquor made from rice, rice koji and Shochu or distilled alcohol), regular | 4.2 | 3.1 | < 0.001 |  |  |  |  |  |  |
|  | Soy sauce, “Koikuchi-shoyu” (common soy sauce) | 17.0 | 13.4 | < 0.001 |  |  |  |  |  |  |
|  | Soy sauce, “Usukuchi-shoyu” (light color soy sauce) | 1.9 | 1.6 | 0.004 |  |  |  |  |  |  |
|  | Vinegar, grain vinegar | 2.2 | 1.8 | 0.047 |  |  |  |  |  |  |
|  | Miso, rice-koji miso, light yellow type | 3.1 | 2.5 | 0.012 |  |  |  |  |  |  |
|  | Miso, rice-koji miso, red type | 5.6 | 4.1 | < 0.001 |  |  |  |  |  |  |
|  | Miso, soybean-koji miso | 5.1 | 4.1 | < 0.001 |  |  |  |  |  |  |
|  | Ponzu sauce, “Mitsukan Ajipon” (soy sauce with citrus juice) | 1.9 | 1.4 | < 0.001 |  |  |  |  |  |  |
| green tea | Green tea, “Sencha” (common grade tea), infusion | 416.1 | 311.9 | < 0.001 |  |  |  |  |  |  |
|  | Green tea, “Hoji-cha” (roasted tea), infusion | 47.3 | 28.8 | 0.001 |  |  |  |  |  |  |
| alcohol | Fermented alcoholic beverage, “Sake”, regular | 37.3 | 21.6 | < 0.001 |  |  |  |  |  |  |

| **Table 7**. Dimensions that describe most of the dietary patterns (women; n =821) ^a^ | | | | |
| --- | --- | --- | --- | --- |
|  |  | Mean in category | Overall mean | P-value |
| Western diet | |  |  |  |
|  | Dim.5 | -0.102 | -5.6E-17 | < 0.001 |
|  | Dim.4 | -0.167 | -3.6E-16 | 0.001 |
|  | Dim.3 | -0.181 | -1.6E-16 | 0.001 |
|  | Dim.2 | -0.561 | 7.1E-17 | < 0.001 |
|  | Dim.1 | -1.099 | 8.0E-17 | < 0.001 |
|  |  |  |  |  |
| Grain-Vegetable-Fruit diet | |  |  |  |
|  | Dim.2 | 2.479 | 7.1E-17 | < 0.001 |
|  | Dim.5 | 0.468 | -5.6E-17 | < 0.001 |
|  | Dim.3 | 0.299 | -1.6E-16 | 0.006 |
|  |  |  |  |  |
| Traditional Japanese diet | |  |  |  |
|  | Dim.1 | 1.580 | 8.0E-17 | < 0.001 |
|  | Dim.4 | 0.221 | -3.6E-16 | 0.001 |
|  | Dim.2 | -0.483 | 7.1E-17 | < 0.001 |
| a | A significance threshold of 0.05 was used to select dimensions that characterized the dietary patterns. | | | |

| **Table 8**. The basic information of each food group to the dimensions that are the most associated with dietary patterns (women; n =821) | | | | | | | | | | | | |
| --- | --- | --- | --- | --- | --- | --- | --- | --- | --- | --- | --- | --- |
| Food groups | | Coordinates | | | | |  | Contributions ^a^ | | | | |
|  |  | Dim.1 | Dim.2 | Dim.3 | Dim.4 | Dim.5 |  | Dim.1 | Dim.2 | Dim.3 | Dim.4 | Dim.5 |
| Cereals | | 0.260 | 0.274 | 0.344 | 0.079 | 0.188 |  | 9.950 | 10.997 | 16.791 | 4.022 | 9.944 |
| Fish & shellfish | | 0.140 | 0.130 | 0.139 | 0.132 | 0.159 |  | 5.349 | 5.205 | 6.778 | 6.670 | 8.427 |
| Red meat | | 0.134 | 0.062 | 0.086 | 0.081 | 0.051 |  | 5.136 | 2.487 | 4.200 | 4.079 | 2.693 |
| White meat | | 0.033 | 0.012 | 0.051 | 0.075 | 0.047 |  | 1.279 | 0.482 | 2.480 | 3.803 | 2.472 |
| Eggs | | 0.045 | 0.004 | 0.009 | 0.057 | 0.050 |  | 1.717 | 0.169 | 0.442 | 2.890 | 2.640 |
| Legumes | | 0.187 | 0.039 | 0.115 | 0.090 | 0.168 |  | 7.144 | 1.564 | 5.610 | 4.551 | 8.888 |
| Vegetable | | 0.246 | 0.584 | 0.107 | 0.105 | 0.138 |  | 9.393 | 23.396 | 5.249 | 5.321 | 7.309 |
| Pickles | | 0.074 | 0.125 | 0.054 | 0.060 | 0.074 |  | 2.836 | 5.000 | 2.650 | 3.022 | 3.928 |
| Tubers | | 0.190 | 0.120 | 0.105 | 0.143 | 0.077 |  | 7.249 | 4.801 | 5.126 | 7.259 | 4.079 |
| Mushroom | | 0.073 | 0.038 | 0.015 | 0.088 | 0.085 |  | 2.778 | 1.509 | 0.752 | 4.434 | 4.476 |
| Seaweeds | | 0.077 | 0.032 | 0.021 | 0.102 | 0.101 |  | 2.955 | 1.299 | 1.015 | 5.183 | 5.351 |
| Fruits | | 0.195 | 0.506 | 0.101 | 0.219 | 0.091 |  | 7.462 | 20.256 | 4.949 | 11.113 | 4.790 |
| Dairy | | 0.037 | 0.051 | 0.191 | 0.100 | 0.046 |  | 1.423 | 2.061 | 9.324 | 5.063 | 2.451 |
| Vegetable oils | | 0.192 | 0.012 | 0.266 | 0.060 | 0.026 |  | 7.324 | 0.497 | 12.980 | 3.037 | 1.370 |
| Seasonings | | 0.235 | 0.149 | 0.255 | 0.141 | 0.187 |  | 8.974 | 5.953 | 12.470 | 7.126 | 9.873 |
| Sweets, sugars & sweeteners | | 0.082 | 0.108 | 0.101 | 0.199 | 0.102 |  | 3.120 | 4.320 | 4.924 | 10.091 | 5.398 |
| Green tea | | 0.143 | 0.019 | 0.004 | 0.027 | 0.048 |  | 5.455 | 0.771 | 0.181 | 1.373 | 2.538 |
| Other tea | | 0.039 | 0.126 | 0.011 | 0.101 | 0.091 |  | 1.475 | 5.051 | 0.528 | 5.131 | 4.800 |
| Coffee | | 0.083 | 0.016 | 0.015 | 0.017 | 0.096 |  | 3.168 | 0.654 | 0.734 | 0.853 | 5.081 |
| Soft drinks | | 0.044 | 0.070 | 0.032 | 0.065 | 0.015 |  | 1.685 | 2.823 | 1.549 | 3.299 | 0.768 |
| Alcohol | | 0.108 | 0.018 | 0.026 | 0.033 | 0.052 |  | 4.128 | 0.706 | 1.267 | 1.680 | 2.724 |
| a | Red values indicate the food groups which contributed more than the expected average contribution (1/21×100%) of all food groups. | | | | | | | | | | | |

| **Table 9**. The basic information of the top 24 (10%) food items to the dimensions that are most associated with dietary patterns (women; n =821) | | | | | | | | | | | | |
| --- | --- | --- | --- | --- | --- | --- | --- | --- | --- | --- | --- | --- |
| Food items | | Coordinates | | | | |  | Contributions ^a^ | | | | |
|  |  | Dim.1 | Dim.2 | Dim.3 | Dim.4 | Dim.5 |  | Dim.1 | Dim.2 | Dim.3 | Dim.4 | Dim.5 |
| Rice, short grain, paddy rice, brown, “meshi” (cooked rice) | | 0.144 | 0.082 | -0.255 | 0.151 | -0.048 |  | 0.545 | 0.187 | 2.188 | 0.802 | 0.083 |
| Rice, short grain, paddy rice, under-milled, “meshi” (cooked rice) | | 0.203 | -0.057 | 0.017 | -0.083 | 0.082 |  | 1.083 | 0.089 | 0.010 | 0.241 | 0.248 |
| Rice, short grain, paddy rice, nonglutinous rice, well-milled, “meshi” (cooked rice) | | 0.248 | 0.080 | 0.430 | 0.054 | 0.121 |  | 1.629 | 0.177 | 6.248 | 0.103 | 0.539 |
| Common wheat, soft flour, first grade | | -0.135 | -0.110 | 0.226 | 0.041 | -0.177 |  | 0.483 | 0.334 | 1.728 | 0.057 | 1.144 |
| Common wheat, bread, white | | -0.195 | -0.046 | -0.180 | -0.024 | -0.120 |  | 1.006 | 0.059 | 1.099 | 0.020 | 0.525 |
| Common wheat, bread, white long roll | | -0.184 | -0.052 | 0.174 | 0.030 | -0.012 |  | 0.897 | 0.074 | 1.019 | 0.032 | 0.006 |
| Common wheat, “Somen and Hiyamugi” (thin wheat noodles), dried noodles, boiled | | 0.006 | 0.478 | -0.124 | 0.058 | 0.072 |  | 0.001 | 6.325 | 0.516 | 0.117 | 0.191 |
| Common wheat, yellow alkaline noodles, steamed noodles | | -0.159 | -0.041 | 0.218 | 0.040 | -0.232 |  | 0.669 | 0.046 | 1.609 | 0.055 | 1.971 |
| Durum wheat, macaroni and spaghetti, dry pasta, boiled | | -0.225 | -0.097 | -0.068 | -0.022 | 0.030 |  | 1.335 | 0.263 | 0.155 | 0.017 | 0.033 |
| Instant Chinese noodles, unseasoned, deep-fried | | -0.058 | -0.154 | -0.016 | 0.007 | 0.289 |  | 0.088 | 0.657 | 0.009 | 0.002 | 3.046 |
| Fish, Pacific saury, with integument, baked | | 0.041 | 0.083 | 0.086 | -0.096 | 0.187 |  | 0.052 | 0.224 | 0.289 | 0.376 | 1.482 |
| Fish, tuna, yellowfin tuna, raw | | 0.084 | -0.017 | 0.137 | -0.102 | 0.229 |  | 0.216 | 0.009 | 0.740 | 0.425 | 2.233 |
| Fish, eel, “Kabayaki” (seasoned and baked fillet) | | -0.019 | 0.131 | -0.157 | 0.019 | 0.021 |  | 0.011 | 0.556 | 0.973 | 0.015 | 0.019 |
| Mollusks, Pacific oyster, cultured, boiled | | 0.153 | -0.191 | -0.001 | -0.033 | -0.008 |  | 0.719 | 1.183 | 0.000 | 0.046 | 0.003 |
| Crustacean, giant tiger prawn, cultured, raw | | -0.134 | -0.095 | 0.207 | 0.014 | 0.031 |  | 0.551 | 0.292 | 1.691 | 0.008 | 0.041 |
| Mollusks, Japanese common squid*, raw [*Syn. short-finned squid] | | -0.059 | -0.025 | 0.157 | -0.086 | -0.052 |  | 0.106 | 0.020 | 0.970 | 0.301 | 0.113 |
| Mollusks, common octopus, boiled | | -0.128 | 0.095 | -0.009 | 0.030 | -0.205 |  | 0.507 | 0.293 | 0.003 | 0.037 | 1.795 |
| Surimi products, “Kanifumi-kamaboko” (imitation crab meat made from surimi) | | -0.015 | 0.075 | 0.001 | 0.270 | 0.081 |  | 0.007 | 0.181 | 0.000 | 2.972 | 0.277 |
| Pork, large type breed, picnic shoulder, lean and fat, raw | | -0.023 | 0.047 | 0.233 | -0.003 | -0.104 |  | 0.017 | 0.072 | 2.128 | 0.000 | 0.458 |
| Pork, sausage, Vienna | | -0.202 | 0.075 | -0.100 | 0.133 | 0.021 |  | 1.253 | 0.182 | 0.389 | 0.723 | 0.019 |
| Chicken, broiler, breast, meat with skin, raw | | -0.158 | -0.057 | 0.197 | 0.016 | -0.069 |  | 0.895 | 0.124 | 1.775 | 0.012 | 0.235 |
| Chicken, ground meat, raw | | 0.050 | -0.026 | 0.084 | 0.271 | 0.148 |  | 0.090 | 0.026 | 0.327 | 3.491 | 1.087 |
| Eggs, hen, whole, raw | | 0.163 | -0.033 | -0.083 | 0.161 | 0.233 |  | 0.934 | 0.039 | 0.313 | 1.214 | 2.634 |
| Eggs, hen, whole, boiled | | -0.149 | -0.059 | 0.054 | 0.190 | 0.011 |  | 0.784 | 0.130 | 0.129 | 1.676 | 0.006 |
| Soybeans, tofu, “Kinugoshi-tofu” (silken tofu) | | 0.217 | 0.007 | -0.070 | -0.065 | -0.124 |  | 1.453 | 0.001 | 0.195 | 0.172 | 0.656 |
| Soybeans, tofu, soft tofu | | -0.037 | 0.070 | 0.063 | -0.027 | 0.269 |  | 0.042 | 0.161 | 0.158 | 0.029 | 3.087 |
| Soybeans, tofu, “Nama-age” (fried slices of drained tofu) | | 0.137 | -0.028 | -0.100 | 0.161 | -0.149 |  | 0.576 | 0.026 | 0.394 | 1.057 | 0.945 |
| Soybeans, natto, “Itohiki-natto” (fermented whole soybean) | | 0.288 | 0.085 | -0.265 | 0.126 | 0.126 |  | 2.569 | 0.232 | 2.769 | 0.645 | 0.675 |
| Kidney beans, “Sayaingen”, immature pods, boiled | | -0.028 | 0.291 | -0.092 | 0.027 | -0.011 |  | 0.012 | 1.344 | 0.164 | 0.014 | 0.003 |
| Okra, pods, boiled | | 0.003 | 0.301 | 0.017 | 0.004 | 0.113 |  | 0.000 | 1.435 | 0.006 | 0.000 | 0.268 |
| Tomatoes, fruit, raw | | 0.086 | 0.308 | -0.113 | 0.134 | -0.051 |  | 0.112 | 1.507 | 0.247 | 0.359 | 0.054 |
| Carrot, regular (European type), root without skin, boiled | | 0.306 | 0.014 | 0.070 | 0.072 | -0.070 |  | 1.421 | 0.003 | 0.095 | 0.104 | 0.102 |
| Sweet peppers, fruit, green, raw | | 0.013 | 0.287 | 0.012 | 0.048 | 0.046 |  | 0.003 | 1.305 | 0.003 | 0.046 | 0.044 |
| Spinach, leaves, all season, boiled | | 0.280 | -0.202 | 0.025 | 0.011 | -0.092 |  | 1.186 | 0.650 | 0.012 | 0.002 | 0.177 |
| Cucumber, fruit, raw | | -0.084 | 0.463 | -0.017 | 0.184 | -0.020 |  | 0.106 | 3.405 | 0.005 | 0.678 | 0.008 |
| Corn, sweet corn, immature kernels, boiled | | -0.034 | 0.283 | -0.031 | 0.010 | 0.172 |  | 0.017 | 1.271 | 0.018 | 0.002 | 0.619 |
| Eggplant*, Japanese type, fruit, boiled [*Syn. Aubergine] | | -0.037 | 0.460 | 0.107 | 0.014 | 0.153 |  | 0.021 | 3.358 | 0.220 | 0.004 | 0.490 |
| Bitter melon, fruit, raw | | 0.032 | 0.364 | 0.030 | -0.147 | 0.056 |  | 0.016 | 2.101 | 0.018 | 0.434 | 0.066 |
| Chinese cabbage, head, boiled | | 0.089 | -0.308 | 0.094 | 0.053 | 0.170 |  | 0.120 | 1.508 | 0.170 | 0.056 | 0.605 |
| Cucumber, fruit, pickles, salted pickles | | 0.010 | 0.208 | -0.023 | 0.004 | 0.011 |  | 0.004 | 1.557 | 0.023 | 0.001 | 0.005 |
| Japanese radishes, Daikon, root, pickles, “Takuan-zuke” (pickled with rice bran and salt), made of salted Daikon | | 0.137 | -0.005 | -0.004 | -0.051 | -0.225 |  | 0.638 | 0.001 | 0.001 | 0.120 | 2.385 |
| Japanese scallion, “Rakkyo”, mature bulb, pickles, sweetened | | 0.177 | 0.263 | 0.092 | -0.252 | -0.159 |  | 1.071 | 2.472 | 0.371 | 2.872 | 1.192 |
| Sweet potato, tuberous root, without skin, raw | | 0.106 | 0.213 | 0.144 | -0.286 | -0.014 |  | 0.336 | 1.424 | 0.797 | 3.242 | 0.008 |
| Sweet potato, tuberous root, without skin, steamed | | 0.037 | -0.043 | -0.017 | 0.172 | 0.189 |  | 0.040 | 0.058 | 0.011 | 1.169 | 1.484 |
| Taro, “Satoimo”, corm, boiled | | 0.391 | -0.132 | 0.091 | -0.168 | 0.042 |  | 4.581 | 0.550 | 0.319 | 1.118 | 0.074 |
| Potatoes, tuber, boiled | | 0.060 | 0.234 | 0.270 | -0.006 | -0.127 |  | 0.108 | 1.715 | 2.788 | 0.001 | 0.665 |
| Konjac, noodles | | 0.060 | -0.073 | 0.162 | 0.174 | 0.159 |  | 0.107 | 0.165 | 1.003 | 1.200 | 1.043 |
| Mushrooms, winter mushrooms*, boiled [*Syn. Enokitake, Enoki] | | 0.164 | -0.100 | -0.039 | 0.129 | 0.270 |  | 0.854 | 0.333 | 0.061 | 0.704 | 3.212 |
| Mushrooms, “Shiitake”, boiled | | 0.153 | -0.047 | 0.001 | 0.204 | 0.084 |  | 0.748 | 0.072 | 0.000 | 1.745 | 0.309 |
| Algae, “Mozuku”, salted products, desalted | | 0.177 | 0.147 | -0.098 | 0.272 | 0.004 |  | 1.082 | 0.791 | 0.423 | 3.403 | 0.001 |
| Algae, “Wakame”, cut and dried | | 0.162 | 0.027 | 0.009 | -0.103 | 0.214 |  | 0.913 | 0.026 | 0.003 | 0.489 | 2.200 |
| Algae, “Wakame”, blanched and salted products, desalted | | 0.118 | 0.061 | 0.109 | 0.122 | -0.230 |  | 0.482 | 0.133 | 0.526 | 0.689 | 2.534 |
| Strawberries, raw | | 0.104 | -0.139 | 0.014 | 0.187 | -0.077 |  | 0.257 | 0.475 | 0.006 | 1.091 | 0.191 |
| Figs, raw | | 0.066 | 0.304 | 0.160 | -0.312 | 0.021 |  | 0.103 | 2.282 | 0.770 | 3.029 | 0.015 |
| Japanese persimmons*, nonastringent, raw [*Syn. Kaki] | | 0.274 | -0.060 | 0.090 | -0.181 | 0.115 |  | 1.770 | 0.090 | 0.242 | 1.025 | 0.428 |
| Watermelon, red flesh type, raw | | -0.074 | 0.347 | -0.054 | 0.021 | 0.020 |  | 0.128 | 2.970 | 0.086 | 0.014 | 0.013 |
| Grapes, raw | | 0.042 | 0.359 | 0.156 | -0.321 | 0.072 |  | 0.042 | 3.181 | 0.734 | 3.209 | 0.171 |
| Muskmelon, greenhouse culture, raw | | -0.073 | 0.225 | -0.095 | 0.012 | 0.024 |  | 0.125 | 1.253 | 0.273 | 0.005 | 0.019 |
| Muskmelon, open culture, green flesh type, raw | | 0.012 | 0.317 | -0.002 | 0.068 | -0.011 |  | 0.003 | 2.484 | 0.000 | 0.146 | 0.004 |
| Peaches, raw | | -0.047 | 0.366 | -0.056 | 0.043 | 0.116 |  | 0.052 | 3.308 | 0.093 | 0.058 | 0.442 |
| Satsuma mandarins, juice sacs, normal ripening type, raw | | 0.253 | -0.277 | 0.060 | -0.071 | 0.137 |  | 1.508 | 1.890 | 0.107 | 0.157 | 0.611 |
| Liquid milk, containing recombined milk, low fat | | 0.052 | -0.032 | -0.252 | 0.001 | -0.008 |  | 0.082 | 0.031 | 2.438 | 0.000 | 0.003 |
| Yogurt, whole milk, unsweetened | | 0.092 | 0.115 | -0.350 | 0.191 | -0.107 |  | 0.255 | 0.414 | 4.696 | 1.440 | 0.470 |
| Yogurt, skimmed, sweetened | | -0.083 | -0.049 | 0.043 | -0.224 | 0.047 |  | 0.205 | 0.077 | 0.071 | 1.997 | 0.092 |
| Vegetable oil, blend | | -0.264 | -0.061 | 0.483 | 0.126 | -0.038 |  | 2.353 | 0.129 | 10.045 | 0.704 | 0.066 |
| Fat spread | | -0.285 | -0.081 | -0.154 | -0.059 | -0.130 |  | 2.736 | 0.229 | 1.022 | 0.155 | 0.789 |
| Dressing, mayonnaise, egg yolk type | | -0.258 | -0.063 | 0.211 | 0.221 | -0.105 |  | 2.235 | 0.138 | 1.913 | 2.178 | 0.515 |
| Compound alcoholic beverage, “Mirin” (sweet liquor made from rice, rice koji and Shochu or distilled alcohol), regular | | 0.237 | 0.141 | 0.108 | 0.078 | 0.022 |  | 1.365 | 0.507 | 0.363 | 0.197 | 0.016 |
| Japanese Worcester sauce, common type | | -0.155 | -0.069 | 0.329 | 0.010 | -0.289 |  | 0.581 | 0.121 | 3.365 | 0.003 | 2.807 |
| Soy sauce, “Koikuchi-shoyu” (common soy sauce) | | 0.341 | 0.180 | 0.319 | 0.182 | 0.085 |  | 2.829 | 0.821 | 3.168 | 1.068 | 0.244 |
| Soy sauce, “Usukuchi-shoyu” (light color soy sauce) | | 0.052 | -0.044 | -0.181 | -0.018 | 0.158 |  | 0.067 | 0.050 | 1.012 | 0.010 | 0.835 |
| Vinegar, grain vinegar | | 0.160 | 0.198 | 0.110 | 0.308 | 0.061 |  | 0.623 | 0.996 | 0.374 | 3.051 | 0.126 |
| Soup stock, beef and vegetable stock | | -0.105 | -0.137 | -0.134 | -0.034 | 0.249 |  | 0.269 | 0.481 | 0.555 | 0.037 | 2.088 |
| Miso, rice-koji miso, red type | | 0.139 | -0.021 | 0.106 | 0.025 | 0.213 |  | 0.468 | 0.012 | 0.351 | 0.021 | 1.519 |
| Miso, soybean-koji miso | | 0.220 | 0.048 | 0.168 | 0.078 | -0.101 |  | 1.176 | 0.058 | 0.877 | 0.194 | 0.342 |
| Cake and pastry, layered cream cake | | -0.023 | -0.053 | 0.122 | 0.007 | 0.180 |  | 0.015 | 0.087 | 0.569 | 0.002 | 1.330 |
| Cake and pastry, Danish pastry | | -0.103 | -0.065 | -0.007 | 0.181 | 0.070 |  | 0.314 | 0.130 | 0.002 | 1.295 | 0.200 |
| Sugars, soft sugars, white | | 0.031 | 0.126 | 0.243 | 0.397 | 0.167 |  | 0.029 | 0.492 | 2.242 | 6.211 | 1.151 |
| Green tea, “Sencha” (common grade tea), infusion | | 0.395 | -0.129 | 0.050 | 0.112 | -0.196 |  | 5.208 | 0.587 | 0.108 | 0.558 | 1.783 |
| Fermented tea, black tea, infusion | | 0.036 | -0.061 | -0.011 | 0.313 | -0.054 |  | 0.042 | 0.129 | 0.005 | 4.304 | 0.133 |
| “Mugi-cha” (roasted barley tea), infusion | | -0.187 | 0.370 | 0.099 | 0.095 | 0.319 |  | 1.158 | 4.750 | 0.416 | 0.393 | 4.657 |
| Milk beverages, coffee flavored | | -0.045 | 0.054 | 0.132 | 0.070 | 0.249 |  | 0.060 | 0.094 | 0.671 | 0.199 | 2.585 |
| Coffee, infusion | | -0.281 | -0.039 | -0.005 | -0.036 | -0.084 |  | 2.379 | 0.049 | 0.001 | 0.051 | 0.291 |
| Coffee, ready-to-drink coffee with milk and sugar, canned | | -0.101 | -0.064 | 0.036 | 0.062 | 0.171 |  | 0.311 | 0.130 | 0.049 | 0.154 | 1.222 |
| Apples, 30 % fruit juice beverage | | 0.027 | 0.220 | 0.060 | -0.270 | -0.038 |  | 0.023 | 1.529 | 0.138 | 2.907 | 0.061 |
| Sports drink, “Pocari Sweat” | | -0.167 | 0.135 | 0.160 | 0.068 | 0.050 |  | 0.839 | 0.572 | 0.988 | 0.184 | 0.104 |
| Fermented alcoholic beverage, “Happoshu” (beer-like beverage with less than 67% malt content) | | -0.220 | -0.009 | -0.008 | 0.063 | 0.126 |  | 1.600 | 0.003 | 0.003 | 0.171 | 0.724 |
| Distilled alcoholic beverage, “Shochu”, distilled through a pot still | | -0.103 | -0.084 | -0.118 | -0.023 | 0.195 |  | 0.350 | 0.245 | 0.592 | 0.024 | 1.743 |
| Shochu highball, “Takohai Lemon Lime” | | -0.205 | -0.070 | -0.008 | -0.093 | -0.026 |  | 1.388 | 0.169 | 0.003 | 0.375 | 0.032 |
| a | Red values indicate the top 24 (10%) food items to each dimension. | | | | | | | | | | | |

| **Table 10**. Food items that compose most of the Western diet (women) | | | | | | | | | | |
| --- | --- | --- | --- | --- | --- | --- | --- | --- | --- | --- |
|  | | Mean consumption volume more than overall mean | | *P*-value |  |  |  | Mean consumption volume less than overall mean | | *P*-value |
|  |  | Mean in category (g/day) | Overall mean (g/day) |  |  |  |  | Mean in category (g/day) | Overall mean (g/day) |  |
| No. of participants | | 387 | 821 |  |  |  |  | 387 | 821 |  |
| Food groups | Food items |  |  |  |  | Food groups | Food items |  |  |  |
| cereals | Common wheat, soft flour, first grade | 3.5 | 3.0 | 0.023 |  | cereals | Common wheat, “Somen and Hiyamugi” (thin wheat noodles), dried noodles, boiled | 2.0 | 6.1 | < 0.001 |
|  | Common wheat, bread, white | 38.7 | 31.9 | < 0.001 |  |  | Rice, short grain, paddy rice, brown, “meshi” (cooked rice) | 1.4 | 4.2 | 0.007 |
|  | Common wheat, bread, white long roll | 3.7 | 2.8 | 0.007 |  |  | Rice, short grain, paddy rice, nonglutinous rice, well-milled, “meshi” (cooked rice) | 189.8 | 221.3 | < 0.001 |
|  | Common wheat, bread, soft rolls | 8.1 | 6.0 | < 0.001 |  |  | Rice, short grain, paddy rice, well-milled, rice with embryo, “meshi” (cooked rice) | 0.0 | 1.3 | 0.015 |
|  | Common wheat, “Udon” (thick wheat noodles), boiled | 24.0 | 21.0 | 0.025 |  | fish & shellfish | Fish, horse mackerel, Japanese Jack mackerel＊, “Hirakiboshi” (salted and semi-dried split), baked [*Syn. horse mackerel] | 0.7 | 1.7 | < 0.001 |
|  | Common wheat, yellow alkaline noodles, boiled | 11.1 | 8.9 | 0.011 |  |  | Fish, salmon and trout, chum salmon, “Shiozake” (salted salmon), raw | 2.1 | 3.1 | 0.001 |
|  | Common wheat, yellow alkaline noodles, steamed noodles | 7.8 | 6.4 | 0.027 |  |  | Fish, Pacific saury, with integument, raw | 0.5 | 1.1 | 0.027 |
|  | Durum wheat, macaroni and spaghetti, dry pasta, boiled | 17.6 | 11.5 | < 0.001 |  |  | Fish, Pacific saury, with integument, baked | 1.5 | 2.8 | 0.001 |
|  | Rice, “Sushi Rice” (with vinegar) | 13.7 | 9.7 | < 0.001 |  |  | Mollusks, Pacific oyster, cultured, boiled | 0.5 | 1.2 | 0.006 |
|  | Instant Chinese noodles, unseasoned, deep-fried | 3.3 | 2.6 | 0.016 |  | white meat | Chicken, broiler, thigh, meat with skin, boiled | 7.0 | 8.5 | 0.017 |
| fish & shellfish | Crustacean, giant tiger prawn, cultured, raw | 3.3 | 2.5 | < 0.001 |  | eggs | Eggs, hen, whole, raw | 0.8 | 1.8 | < 0.001 |
| red meat | Beef, ground meat, raw | 2.3 | 1.7 | 0.003 |  | soybean & soybean products | Soybeans, “Budo-mame” (beans cooked with sugar and salt) | 0.9 | 1.4 | 0.019 |
|  | Pork, large type breed, loin, lean and fat, boiled | 3.6 | 2.7 | 0.008 |  |  | Soybeans, tofu, “Momen-tofu” (regular tofu) | 9.9 | 14.8 | < 0.001 |
|  | Pork, ground meat, raw | 3.3 | 2.7 | 0.029 |  |  | Soybeans, tofu, “Kinugoshi-tofu” (silken tofu) | 4.7 | 7.2 | < 0.001 |
|  | Pork, bacon | 2.6 | 2.0 | < 0.001 |  |  | Soybeans, tofu, soft tofu | 4.8 | 6.5 | 0.007 |
|  | Pork, sausage, Vienna | 5.6 | 4.7 | 0.008 |  |  | Soybeans, tofu, “Nama-age” (fried slices of drained tofu) | 2.2 | 2.9 | 0.034 |
|  | Beef and pork mix, ground meat, raw, 7:3 | 1.9 | 1.4 | 0.039 |  |  | Soybeans, tofu, “Abura-age” (fried thin slices of pressed tofu), uncooked | 2.5 | 2.9 | 0.010 |
| white meat | Chicken, broiler, breast, meat with skin, raw | 2.2 | 1.6 | 0.013 |  |  | Soybeans, natto, “Itohiki-natto” (fermented whole soybean) | 3.3 | 5.8 | < 0.001 |
| eggs | Eggs, hen, whole, boiled | 34.5 | 32.7 | 0.018 |  | vegetable | Kidney beans, “Sayaingen”, immature pods, boiled | 1.2 | 1.8 | 0.009 |
| vegetable | Bean sprouts, mung bean sprouts, boiled | 9.7 | 8.3 | 0.027 |  |  | Peas, snap peas, immature pods, raw | 0.4 | 1.0 | 0.007 |
| pickles | Chinese cabbage, head, pickles, Kimchi | 1.9 | 1.4 | 0.018 |  |  | Okra, pods, boiled | 0.5 | 1.1 | < 0.001 |
| fruits | Oranges, Valencia, straight fruit juice | 3.0 | 1.8 | 0.037 |  |  | Pumpkin and squash, winter squash*, fruit, boiled [*Syn. Pumpkin] | 6.8 | 10.1 | < 0.001 |
|  | Grapefruit, white flesh type, juice sacs, raw | 4.9 | 3.5 | 0.048 |  |  | Cucumber, fruit, raw | 11.8 | 15.3 | < 0.001 |
| dairy & dairy products | Yogurt, skimmed, sweetened | 18.2 | 15.2 | 0.005 |  |  | Edible burdock, root, boiled | 2.6 | 3.6 | < 0.001 |
|  | Cheeses, processed | 3.7 | 3.0 | < 0.001 |  |  | Spinach mustard, “Komatsuna”, leaves, boiled | 2.9 | 4.1 | 0.005 |
| vegetable oils | Vegetable oil, blend | 4.8 | 4.4 | 0.003 |  |  | Japanese radishes, Daikon, root without skin, raw | 5.2 | 7.8 | < 0.001 |
|  | Fat spread | 2.4 | 1.6 | < 0.001 |  |  | Japanese radishes, Daikon, root without skin, boiled | 10.6 | 13.2 | 0.003 |
|  | Dressing, mayonnaise, egg yolk type | 3.5 | 2.9 | < 0.001 |  |  | Japanese radishes, Daikon, “Kiriboshi-daikon” (cut and dried Daikon root), raw | 1.6 | 2.7 | 0.004 |
| seasonings | Soup stock, beef and vegetable stock | 3.0 | 2.1 | < 0.001 |  |  | Onions, bulb, boiled | 22.4 | 26.0 | < 0.001 |
|  | Tomato products, ketchup | 2.1 | 1.7 | 0.001 |  |  | Corn, sweet corn, immature kernels, boiled | 0.6 | 1.6 | 0.009 |
| sweets, sugars & sweeteners | Cake and pastry, sponge cake | 1.7 | 1.3 | 0.038 |  |  | Tomatoes, fruit, raw | 16.4 | 23.5 | < 0.001 |
|  | Cake and pastry, doughnuts, cake-type | 2.1 | 1.4 | 0.003 |  |  | Eggplant*, Japanese type, fruit, boiled [*Syn. Aubergine] | 6.4 | 10.2 | < 0.001 |
| coffee | Coffee, infusion | 137.2 | 103.8 | < 0.001 |  |  | Bitter melon, fruit, raw | 0.6 | 1.7 | < 0.001 |
|  | Coffee, instant coffee, granules | 1.7 | 1.4 | < 0.001 |  |  | Carrot, regular (European type), root without skin, boiled | 11.5 | 14.7 | < 0.001 |
|  | Coffee, ready-to-drink coffee with milk and sugar, canned | 7.2 | 5.1 | 0.022 |  |  | Welsh onions, “Nebuka-negi” (large variety, blanching cultivation), leaves, raw | 2.2 | 2.7 | 0.048 |
| alcohol | Fermented alcoholic beverage, beer, pale | 21.7 | 15.0 | 0.021 |  |  | Welsh onions, “Ha-negi” (large variety, green), leaves, raw | 3.3 | 4.1 | 0.002 |
|  | Fermented alcoholic beverage, “Happoshu” (beer-like beverage with less than 67% malt content) | 39.1 | 27.2 | 0.003 |  |  | Sweet peppers, fruit, green, raw | 2.7 | 3.7 | < 0.001 |
|  | Distilled alcoholic beverage, “Shochu”, distilled through a pot still | 5.1 | 2.5 | 0.003 |  |  | Spinach, leaves, all season, boiled | 8.0 | 10.2 | 0.001 |
|  | Shochu highball, “Takohai Lemon Lime” | 5.8 | 3.0 | 0.003 |  |  | East Indian lotus root, rhizome, boiled | 1.6 | 2.1 | 0.022 |
|  |  |  |  |  |  | pickles | Cucumber, fruit, pickles, salted pickles | 0.6 | 1.3 | 0.003 |
|  |  |  |  |  |  |  | Japanese radishes, Daikon, root, pickles, “Takuan-zuke” (pickled with rice bran and salt), made of sun-dried Daikon | 0.7 | 1.1 | 0.048 |
|  |  |  |  |  |  |  | Chinese cabbage, head, pickles, salted pickles | 0.6 | 1.1 | 0.012 |
|  |  |  |  |  |  |  | Japanese scallion, “Rakkyo”, mature bulb, pickles, sweetened | 0.3 | 1.0 | < 0.001 |
|  |  |  |  |  |  | tubers | Konjac, block, made from fine powder | 2.9 | 3.7 | 0.008 |
|  |  |  |  |  |  |  | Sweet potato, tuberous root, without skin, raw | 0.8 | 1.6 | 0.006 |
|  |  |  |  |  |  |  | Sweet potato, tuberous root, without skin, baked | 0.5 | 1.2 | 0.015 |
|  |  |  |  |  |  |  | Taro, “Satoimo”, corm, boiled | 2.8 | 5.8 | < 0.001 |
|  |  |  |  |  |  |  | Potatoes, tuber, boiled | 16.8 | 20.1 | < 0.001 |
|  |  |  |  |  |  |  | Yam, Chinese yam, “Nagaimo”, tuberous root, raw | 1.9 | 2.9 | 0.009 |
|  |  |  |  |  |  | mushroom | Mushrooms, winter mushrooms*, boiled [*Syn. Enokitake, Enoki] | 2.3 | 2.8 | 0.008 |
|  |  |  |  |  |  |  | Mushrooms, “Shiitake”, boiled | 1.1 | 1.7 | < 0.001 |
|  |  |  |  |  |  |  | Mushrooms, “Shiitake”, dried, boiled | 0.7 | 1.1 | < 0.001 |
|  |  |  |  |  |  |  | Mushrooms, beech mushrooms, boiled | 2.4 | 2.9 | 0.027 |
|  |  |  |  |  |  | seaweeds | Algae, “Hijiki”, dried | 1.3 | 2.6 | < 0.001 |
|  |  |  |  |  |  |  | Algae, “Mozuku”, salted products, desalted | 0.5 | 1.7 | < 0.001 |
|  |  |  |  |  |  |  | Algae, “Wakame”, cut and dried | 3.8 | 5.5 | < 0.001 |
|  |  |  |  |  |  |  | Algae, “Wakame”, blanched and salted products, desalted | 1.8 | 2.4 | 0.006 |
|  |  |  |  |  |  | fruits | Figs, raw | 0.8 | 1.8 | 0.030 |
|  |  |  |  |  |  |  | Citrus, “Iyo”, juice sacs | 1.0 | 2.0 | 0.035 |
|  |  |  |  |  |  |  | Satsuma mandarins, juice sacs, normal ripening type, raw | 9.5 | 14.4 | 0.002 |
|  |  |  |  |  |  |  | Japanese persimmons*, nonastringent, raw [*Syn. Kaki] | 3.6 | 7.1 | 0.001 |
|  |  |  |  |  |  |  | Watermelon, red flesh type, raw | 4.2 | 7.9 | 0.009 |
|  |  |  |  |  |  |  | Grapes, raw | 1.5 | 4.8 | < 0.001 |
|  |  |  |  |  |  |  | Muskmelon, open culture, green flesh type, raw | 0.8 | 2.6 | 0.001 |
|  |  |  |  |  |  |  | Peaches, raw | 0.3 | 2.1 | < 0.001 |
|  |  |  |  |  |  |  | Apples, without skin, raw | 14.6 | 18.5 | 0.005 |
|  |  |  |  |  |  | seasonings | Compound alcoholic beverage, “Mirin” (sweet liquor made from rice, rice koji and Shochu or distilled alcohol), regular | 1.8 | 2.5 | < 0.001 |
|  |  |  |  |  |  |  | Soy sauce, “Koikuchi-shoyu” (common soy sauce) | 7.9 | 10.6 | < 0.001 |
|  |  |  |  |  |  |  | Vinegar, grain vinegar | 1.1 | 1.8 | < 0.001 |
|  |  |  |  |  |  |  | Vinegar, rice vinegar | 0.5 | 1.0 | 0.001 |
|  |  |  |  |  |  |  | Miso, rice-koji miso, light yellow type | 1.6 | 2.0 | 0.009 |
|  |  |  |  |  |  |  | Miso, rice-koji miso, red type | 2.5 | 3.2 | < 0.001 |
|  |  |  |  |  |  |  | Miso, soybean-koji miso | 2.0 | 2.8 | < 0.001 |
|  |  |  |  |  |  |  | Ponzu sauce, “Mitsukan Ajipon” (soy sauce with citrus juice) | 1.2 | 1.5 | 0.021 |
|  |  |  |  |  |  |  | Cooking Sake, “Mitsukan Cooking Sake” | 0.8 | 1.2 | 0.003 |
|  |  |  |  |  |  | sweets, sugars & sweeteners | Sugars, soft sugars, white | 3.6 | 4.1 | 0.001 |
|  |  |  |  |  |  | green tea | Green tea, “Sencha” (common grade tea), infusion | 216.6 | 299.9 | < 0.001 |
|  |  |  |  |  |  | other tea | “Mugi-cha” (roasted barley tea), infusion | 103.1 | 124.8 | 0.025 |

| **Table 11**. Food items that compose most of the Grain-Vegetable-Fruit diet (women) | | | | | | | | | | |
| --- | --- | --- | --- | --- | --- | --- | --- | --- | --- | --- |
|  | | Mean consumption volume more than overall mean | | *P*-value |  |  |  | Mean consumption volume less than overall mean | | *P*-value |
|  |  | Mean in category (g/day) | Overall mean (g/day) |  |  |  |  | Mean in category (g/day) | Overall mean (g/day) |  |
| No. of participants | | 144 | 821 |  |  |  |  | 144 | 821 |  |
| Food groups | Food items |  |  |  |  | Food groups | Food items |  |  |  |
| cereals | Common wheat, “Somen and Hiyamugi” (thin wheat noodles), dried noodles, boiled | 26.1 | 6.1 | < 0.001 |  | cereals | Common wheat, bread, soft rolls | 3.7 | 6.0 | 0.043 |
|  | Rice, short grain, paddy rice, nonglutinous rice, well-milled, “meshi” (cooked rice) | 247.8 | 221.3 | 0.001 |  |  | Common wheat, “Udon” (thick wheat noodles), boiled | 12.7 | 21.0 | 0.002 |
|  | Buckwheat, dried noodles, boiled | 5.4 | 2.7 | 0.017 |  |  | Durum wheat, macaroni and spaghetti, dry pasta, boiled | 5.8 | 11.5 | 0.005 |
| fish & shellfish | Fish, eel, “Kabayaki” (seasoned and baked fillet) | 2.2 | 1.1 | 0.007 |  |  | Rice, glutinous rice products, rice cake | 1.5 | 3.9 | 0.020 |
|  | Fish, salmon and trout, chum salmon, “Shiozake” (salted salmon), raw | 4.7 | 3.1 | 0.006 |  |  | Rice, “Sushi Rice” (with vinegar) | 5.6 | 9.7 | 0.030 |
|  | Fish, Pacific saury, with integument, baked | 5.3 | 2.8 | 0.002 |  |  | Instant Chinese noodles, unseasoned, deep-fried | 1.2 | 2.6 | 0.023 |
|  | Mollusks, common octopus, boiled | 2.2 | 1.3 | 0.022 |  | fish & shellfish | Fish, yellowtail*, mature, raw [*Syn. five-ray yellowtail] | 0.2 | 1.9 | 0.001 |
| red meat | Pork, large type breed, belly, lean and fat, raw | 4.3 | 2.9 | 0.041 |  |  | Mollusks, Pacific oyster, cultured, boiled | 0.0 | 1.2 | 0.021 |
| soybean & soybean products | Soybeans, tofu, “Momen-tofu” (regular tofu) | 23.2 | 14.8 | < 0.001 |  | vegetable | Cabbage, common, head, boiled | 9.7 | 13.5 | 0.027 |
|  | Soybeans, tofu, soft tofu | 11.6 | 6.5 | < 0.001 |  |  | Japanese radishes, Daikon, root without skin, boiled | 7.5 | 13.2 | 0.001 |
| vegetable | Kidney beans, “Sayaingen”, immature pods, boiled | 4.6 | 1.8 | < 0.001 |  |  | Welsh onions, “Nebuka-negi” (large variety, blanching cultivation), leaves, raw | 1.4 | 2.7 | 0.011 |
|  | Okra, pods, boiled | 3.8 | 1.1 | < 0.001 |  |  | Chinese cabbage, head, boiled | 2.3 | 9.6 | < 0.001 |
|  | Pumpkin and squash, winter squash*, fruit, boiled [*Syn. Pumpkin] | 17.8 | 10.1 | < 0.001 |  |  | Broccoli, inflorescence, boiled | 1.3 | 6.3 | < 0.001 |
|  | Cucumber, fruit, raw | 35.4 | 15.3 | < 0.001 |  |  | Spinach, leaves, all season, boiled | 3.4 | 10.2 | < 0.001 |
|  | Onions, bulb, boiled | 32.1 | 26.0 | 0.001 |  | pickles | Japanese radishes, Daikon, root, pickles, “Takuan-zuke” (pickled with rice bran and salt), made of sun-dried Daikon | 0.3 | 1.1 | 0.037 |
|  | Chinese preserving melon, fruit, boiled | 4.5 | 1.5 | < 0.001 |  |  | Chinese cabbage, head, pickles, salted pickles | 0.2 | 1.1 | 0.023 |
|  | Corn, sweet corn, immature kernels, boiled | 6.6 | 1.6 | < 0.001 |  | tubers | Taro, “Satoimo”, corm, boiled | 2.2 | 5.8 | 0.001 |
|  | Tomatoes, fruit, raw | 36.4 | 23.5 | < 0.001 |  | fruits | Strawberries, raw | 2.1 | 4.5 | 0.022 |
|  | Tomatoes, cherry tomatoes, fruit, raw | 8.5 | 5.3 | 0.004 |  |  | Satsuma mandarins, segments, normal ripening type, raw | 1.3 | 4.8 | 0.038 |
|  | Eggplant*, Japanese type, fruit, boiled [*Syn. Aubergine] | 29.6 | 10.2 | < 0.001 |  |  | Satsuma mandarins, juice sacs, normal ripening type, raw | 2.0 | 14.4 | < 0.001 |
|  | Bitter melon, fruit, raw | 6.8 | 1.7 | < 0.001 |  |  | Japanese persimmons*, nonastringent, raw [*Syn. Kaki] | 2.9 | 7.1 | 0.047 |
|  | Sweet peppers, fruit, green, raw | 7.2 | 3.7 | < 0.001 |  |  | Apples, without skin, raw | 11.1 | 18.5 | 0.008 |
|  | Lettuce, head lettuce, crisp type, soil culture, head, raw | 12.3 | 8.5 | 0.001 |  | seasonings | Soup stock, beef and vegetable stock | 1.1 | 2.1 | 0.049 |
| pickles | Cucumber, fruit, pickles, salted pickles | 3.5 | 1.3 | < 0.001 |  |  | Stew cream (mixed) | 0.5 | 1.2 | 0.041 |
|  | Japanese scallion, “Rakkyo”, mature bulb, pickles, sweetened | 2.3 | 1.0 | < 0.001 |  | sweets, sugars & sweeteners | Chinese style steamed bun, stuffed with meat and vegetable | 0.0 | 1.2 | 0.027 |
| tubers | Sweet potato, tuberous root, without skin, raw | 3.2 | 1.6 | 0.009 |  |  | Biscuits, soft biscuits | 0.4 | 1.1 | 0.017 |
|  | Potatoes, tuber, boiled | 29.2 | 20.1 | < 0.001 |  | green tea | Green tea, “Sencha” (common grade tea), infusion | 173.1 | 299.9 | < 0.001 |
| fruits | Figs, raw | 6.9 | 1.8 | < 0.001 |  |  | Green tea, “Hoji-cha” (roasted tea), infusion | 15.8 | 36.6 | 0.034 |
|  | Watermelon, red flesh type, raw | 30.3 | 7.9 | < 0.001 |  | coffee | Coffee, instant coffee, granules | 1.0 | 1.4 | 0.028 |
|  | Pears, sand pears*, raw [*Syn. Nashi pears] | 14.3 | 6.4 | < 0.001 |  |  |  |  |  |  |
|  | Grapes, raw | 19.5 | 4.8 | < 0.001 |  |  |  |  |  |  |
|  | Muskmelon, greenhouse culture, raw | 4.5 | 1.2 | < 0.001 |  |  |  |  |  |  |
|  | Muskmelon, open culture, green flesh type, raw | 11.6 | 2.6 | < 0.001 |  |  |  |  |  |  |
|  | Peaches, raw | 11.0 | 2.1 | < 0.001 |  |  |  |  |  |  |
| dairy & dairy products | Liquid milk, whole milk | 102.3 | 77.4 | 0.001 |  |  |  |  |  |  |
| seasonings | Compound alcoholic beverage, “Mirin” (sweet liquor made from rice, rice koji and Shochu or distilled alcohol), regular | 3.4 | 2.5 | < 0.001 |  |  |  |  |  |  |
|  | Soy sauce, “Koikuchi-shoyu” (common soy sauce) | 13.1 | 10.6 | < 0.001 |  |  |  |  |  |  |
|  | Vinegar, grain vinegar | 2.8 | 1.8 | < 0.001 |  |  |  |  |  |  |
|  | Japanese noodle soup, non-concentrated (soy sauce base) | 4.9 | 2.6 | < 0.001 |  |  |  |  |  |  |
|  | Japanese noodle soup, triple-concentrated (soy sauce base) | 4.7 | 3.7 | 0.033 |  |  |  |  |  |  |
|  | Dressing, soy sauce based, fat-free | 1.7 | 1.2 | 0.032 |  |  |  |  |  |  |
|  | Roux, Japanese curry roux, instant | 1.6 | 1.1 | 0.043 |  |  |  |  |  |  |
| sweets, sugars & sweeteners | Sugars, soft sugars, white | 5.6 | 4.1 | < 0.001 |  |  |  |  |  |  |
|  | Honey | 1.7 | 1.1 | 0.020 |  |  |  |  |  |  |
|  | Ice cream, lacto-ice, regular (milk solids ≥ 3%, main lipid: vegetable fat) | 6.2 | 2.9 | < 0.001 |  |  |  |  |  |  |
| other tea | Fermented tea, Oolong tea, infusion | 126.6 | 88.3 | 0.024 |  |  |  |  |  |  |
|  | “Mugi-cha” (roasted barley tea), infusion | 339.9 | 124.8 | < 0.001 |  |  |  |  |  |  |
| coffee | Milk beverages, coffee flavored | 4.1 | 1.5 | 0.016 |  |  |  |  |  |  |
| soft drinks | Apples, 30 % fruit juice beverage | 3.5 | 1.3 | 0.004 |  |  |  |  |  |  |
|  | Carbonated beverage, clear soda | 5.3 | 1.7 | < 0.001 |  |  |  |  |  |  |
|  | Sports drink, “Pocari Sweat” | 18.4 | 8.4 | 0.001 |  |  |  |  |  |  |
| alcohol | Cocktail, “Screwdriver” (vodka with orange juice) | 5.4 | 1.4 | 0.034 |  |  |  |  |  |  |

| **Table 12**. Food items that compose most of the Traditional Japanese diet (women) | | | | | | | | | | |
| --- | --- | --- | --- | --- | --- | --- | --- | --- | --- | --- |
|  | | Mean consumption volume more than overall mean | | *P*-value |  |  |  | Mean consumption volume less than overall mean | | *P*-value |
|  |  | Mean in category (g/day) | Overall mean (g/day) |  |  |  |  | Mean in category (g/day) | Overall mean (g/day) |  |
| No. of participants | | 290 | 821 |  |  |  |  | 290 | 821 |  |
| Food groups | Food items |  |  |  |  | Food groups | Food items |  |  |  |
| cereals | Rice, short grain, paddy rice, brown, "meshi" (cooked rice) | 8.4 | 4.2 | 0.001 |  | cereals | Common wheat, bread, white | 24.6 | 31.9 | < 0.001 |
|  | Rice, short grain, paddy rice, under-milled, “meshi" (cooked rice) | 2.4 | 1.1 | 0.010 |  |  | Common wheat, bread, white long roll | 1.4 | 2.8 | 0.001 |
|  | Rice, short grain, paddy rice, nonglutinous rice, well-milled, "meshi" (cooked rice) | 250.2 | 221.3 | < 0.001 |  |  | Common wheat, bread, soft rolls | 4.2 | 6.0 | 0.010 |
| fish & shellfish | Fish, horse mackerel, Japanese Jack mackerel＊, "Hirakiboshi" (salted and semi-dried split), baked［*Syn. horse mackerel］ | 3.0 | 1.7 | < 0.001 |  |  | Common wheat, "Somen and Hiyamugi" (thin wheat noodles), dried noodles, boiled | 1.7 | 6.1 | < 0.001 |
|  | Fish, yellowtail*, mature, raw［*Syn. five-ray yellowtail］ | 3.0 | 1.9 | 0.002 |  |  | Common wheat, yellow alkaline noodles, boiled | 6.2 | 8.9 | 0.016 |
|  | Mollusks, short-necked clam*, raw［*Syn. baby-neck clam, Manila clam, Japanese littleneck］ | 2.2 | 1.6 | 0.018 |  |  | Durum wheat, macaroni and spaghetti, dry pasta, boiled | 6.1 | 11.5 | < 0.001 |
|  | Mollusks, Pacific oyster, cultured, boiled | 2.6 | 1.2 | < 0.001 |  |  | Buckwheat, dried noodles, boiled | 1.1 | 2.7 | 0.023 |
|  | Surimi products, "Satsuma-age" (fried surimi) | 4.0 | 2.9 | 0.007 |  |  | Rice, “Sushi Rice” (with vinegar) | 6.4 | 9.7 | 0.006 |
| red meat | Pork, large type breed, inside ham, without subcutaneous fat, boiled | 4.4 | 3.2 | 0.006 |  | fish & shellfish | Fish, eel, "Kabayaki" (seasoned and baked fillet) | 0.5 | 1.1 | 0.032 |
| white meat | Chicken, broiler, thigh, meat with skin, boiled | 11.0 | 8.5 | 0.002 |  |  | Crustacean, giant tiger prawn, cultured, raw | 1.8 | 2.5 | 0.013 |
| eggs | Eggs, hen, whole, raw | 3.1 | 1.8 | < 0.001 |  |  | Mollusks, common octopus, boiled | 0.6 | 1.3 | 0.014 |
| soybean & soybean products | Soybeans, "Budo-mame" (beans cooked with sugar and salt) | 2.5 | 1.4 | < 0.001 |  | red meat | Pork, large type breed, loin, lean and fat, boiled | 1.4 | 2.7 | 0.005 |
|  | Soybeans, tofu, "Momen-tofu" (regular tofu) | 17.1 | 14.8 | 0.045 |  |  | Pork, large type breed, belly, lean and fat, raw | 1.8 | 2.9 | 0.006 |
|  | Soybeans, tofu, "Kinugoshi-tofu" (silken tofu) | 11.5 | 7.2 | < 0.001 |  |  | Pork, ham, loin | 3.1 | 3.9 | 0.013 |
|  | Soybeans, tofu, "Nama-age" (fried slices of drained tofu) | 4.3 | 2.9 | 0.002 |  |  | Pork, bacon | 1.4 | 2.0 | 0.007 |
|  | Soybeans, tofu, "Abura-age" (fried thin slices of pressed tofu), uncooked | 3.8 | 2.9 | 0.001 |  |  | Pork, sausage, Vienna | 3.3 | 4.7 | < 0.001 |
|  | Soybeans, natto, "Itohiki-natto" (fermented whole soybean) | 8.7 | 5.8 | < 0.001 |  | white meat | Chicken, broiler, breast, meat with skin, raw | 0.9 | 1.6 | 0.041 |
| vegetable | Peas, snap peas, immature pods, raw | 2.2 | 1.0 | < 0.001 |  | eggs | Eggs, hen, whole, boiled | 30.5 | 32.7 | 0.025 |
|  | Edible burdock, root, boiled | 5.6 | 3.6 | < 0.001 |  | vegetable | Kidney beans, "Sayaingen", immature pods, boiled | 1.2 | 1.8 | 0.034 |
|  | Spinach mustard, "Komatsuna", leaves, boiled | 6.4 | 4.1 | < 0.001 |  |  | Okra, pods, boiled | 0.5 | 1.1 | 0.006 |
|  | Japanese radishes, Daikon, leaves, boiled | 2.5 | 1.4 | 0.001 |  |  | Cucumber, fruit, raw | 10.1 | 15.3 | < 0.001 |
|  | Japanese radishes, Daikon, root without skin, raw | 11.8 | 7.8 | < 0.001 |  |  | Chinese preserving melon, fruit, boiled | 0.2 | 1.5 | 0.008 |
|  | Japanese radishes, Daikon, root without skin, boiled | 19.5 | 13.2 | < 0.001 |  |  | Corn, sweet corn, immature kernels, boiled | 0.5 | 1.6 | 0.027 |
|  | Japanese radishes, Daikon, "Kiriboshi-daikon" (cut and dried Daikon root), raw | 4.8 | 2.7 | < 0.001 |  |  | Eggplant*, Japanese type, fruit, boiled［*Syn. Aubergine］ | 5.7 | 10.2 | < 0.001 |
|  | Rape, stems and leaves, boiled | 2.0 | 1.1 | 0.007 |  |  | Bitter melon, fruit, raw | 0.6 | 1.7 | 0.001 |
|  | Carrot, regular (European type), root without skin, boiled | 19.8 | 14.7 | < 0.001 |  |  | Bean sprouts, mung bean sprouts, boiled | 6.5 | 8.3 | 0.022 |
|  | Welsh onions, "Nebuka-negi" (large variety, blanching cultivation), leaves, raw | 3.9 | 2.7 | < 0.001 |  |  | Lettuce, head lettuce, crisp type, soil culture, head, raw | 7.0 | 8.5 | 0.030 |
|  | Welsh onions, "Ha-negi" (large variety, green), leaves, raw | 5.5 | 4.1 | < 0.001 |  | fruits | Figs, raw | 0.5 | 1.8 | 0.022 |
|  | Chinese cabbage, head, boiled | 13.5 | 9.6 | < 0.001 |  |  | Oranges, Valencia, straight fruit juice | 0.3 | 1.8 | 0.038 |
|  | Broccoli, inflorescence, boiled | 9.4 | 6.3 | < 0.001 |  |  | Grapefruit, white flesh type, juice sacs, raw | 1.7 | 3.5 | 0.049 |
|  | Spinach, leaves, all season, boiled | 16.4 | 10.2 | < 0.001 |  |  | Watermelon, red flesh type, raw | 1.7 | 7.9 | 0.001 |
|  | East Indian lotus root, rhizome, boiled | 3.2 | 2.1 | < 0.001 |  |  | Grapes, raw | 2.0 | 4.8 | 0.009 |
| pickles | Japanese radishes, Daikon, root, pickles, "Takuan-zuke" (pickled with rice bran and salt), made of salted Daikon | 2.3 | 1.8 | 0.049 |  |  | Muskmelon, greenhouse culture, raw | 0.0 | 1.2 | 0.010 |
|  | Japanese radishes, Daikon, root, pickles, "Takuan-zuke" (pickled with rice bran and salt), made of sun-dried Daikon | 1.9 | 1.1 | < 0.001 |  |  | Muskmelon, open culture, green flesh type, raw | 0.7 | 2.6 | 0.006 |
|  | Chinese cabbage, head, pickles, salted pickles | 2.2 | 1.1 | < 0.001 |  |  | Peaches, raw | 0.0 | 2.1 | < 0.001 |
| tubers | Konjac, block, made from fine powder | 5.2 | 3.7 | < 0.001 |  | dairy & dairy products | Liquid milk, whole milk | 67.4 | 77.4 | 0.027 |
|  | Konjac, noodles | 2.2 | 1.5 | 0.003 |  |  | Yogurt, skimmed, sweetened | 12.3 | 15.2 | 0.043 |
|  | Sweet potato, tuberous root, without skin, baked | 2.5 | 1.2 | < 0.001 |  |  | Cheeses, processed | 2.3 | 3.0 | 0.021 |
|  | Taro, "Satoimo", corm, boiled | 11.6 | 5.8 | < 0.001 |  | vegetable oils | Vegetable oil, blend | 3.6 | 4.4 | < 0.001 |
|  | Yam, Chinese yam, "Nagaimo", tuberous root, raw | 4.0 | 2.9 | 0.017 |  |  | Fat spread | 0.7 | 1.6 | < 0.001 |
| mushroom | Mushrooms, winter mushrooms*, boiled［*Syn. Enokitake, Enoki］ | 4.0 | 2.8 | < 0.001 |  |  | Dressing, mayonnaise, egg yolk type | 2.3 | 2.9 | 0.002 |
|  | Mushrooms, "Shiitake", boiled | 2.5 | 1.7 | < 0.001 |  | seasonings | Soup stock, beef and vegetable stock | 1.4 | 2.1 | 0.028 |
|  | Mushrooms, "Shiitake", dried, boiled | 1.4 | 1.1 | 0.035 |  |  | Tomato products, ketchup | 1.3 | 1.7 | 0.028 |
|  | Mushrooms, beech mushrooms, boiled | 3.6 | 2.9 | 0.007 |  | sweets, sugars & sweeteners | Ice cream, lacto-ice, regular (milk solids ≥ 3%, main lipid: vegetable fat) | 1.9 | 2.9 | 0.049 |
| seaweeds | Algae, "Hijiki", dried | 3.7 | 2.6 | 0.006 |  |  | Cake and pastry, doughnuts, cake-type | 0.8 | 1.4 | 0.030 |
|  | Algae, "Mozuku", salted products, desalted | 3.0 | 1.7 | < 0.001 |  | other tea | Fermented tea, Oolong tea, infusion | 52.8 | 88.3 | 0.001 |
|  | Algae, "Wakame", cut and dried | 7.2 | 5.5 | 0.001 |  |  | "Mugi-cha" (roasted barley tea), infusion | 47.0 | 124.8 | < 0.001 |
|  | Algae, “Wakame", blanched and salted products, desalted | 3.1 | 2.4 | 0.005 |  | coffee | Coffee, infusion | 59.4 | 103.8 | < 0.001 |
| fruits | Strawberries, raw | 7.0 | 4.5 | < 0.001 |  |  | Coffee, instant coffee, granules | 1.2 | 1.4 | 0.036 |
|  | Citrus, "Iyo", juice sacs | 4.2 | 2.0 | 0.001 |  | soft drinks | Carbonated beverage, fruit flavored and colored drink | 0.0 | 1.0 | 0.029 |
|  | Satsuma mandarins, segments, normal ripening type, raw | 8.5 | 4.8 | < 0.001 |  |  | Carbonated beverage, clear soda | 0.2 | 1.7 | 0.022 |
|  | Satsuma mandarins, juice sacs, normal ripening type, raw | 27.2 | 14.4 | < 0.001 |  |  | Sports drink, “Pocari Sweat” | 3.2 | 8.4 | 0.007 |
|  | Japanese persimmons*, nonastringent, raw［*Syn. Kaki］ | 13.8 | 7.1 | < 0.001 |  | alcohol | Fermented alcoholic beverage, beer, pale | 4.6 | 15.0 | 0.006 |
|  | Citrus, "Tangor", juice sacs, raw | 2.6 | 1.3 | 0.004 |  |  | Fermented alcoholic beverage, "Happoshu" (beer-like beverage with less than 67% malt content) | 13.2 | 27.2 | 0.007 |
|  | Citrus, "Hassaku", juice sacs, raw | 3.4 | 1.7 | 0.004 |  |  | Distilled alcoholic beverage, "Shochu", distilled through a pot still | 0.1 | 2.5 | 0.023 |
|  | Apples, without skin, raw | 27.4 | 18.5 | < 0.001 |  |  | Shochu highball, “Takohai Lemon Lime” | 0.2 | 3.0 | 0.027 |
| seasonings | Compound alcoholic beverage, "Mirin" (sweet liquor made from rice, rice koji and Shochu or distilled alcohol), regular | 3.0 | 2.5 | 0.001 |  |  |  |  |  |  |
|  | Soy sauce, "Koikuchi-shoyu" (common soy sauce) | 12.8 | 10.6 | < 0.001 |  |  |  |  |  |  |
|  | Vinegar, grain vinegar | 2.2 | 1.8 | 0.008 |  |  |  |  |  |  |
|  | Miso, rice-koji miso, light yellow type | 2.5 | 2.0 | 0.004 |  |  |  |  |  |  |
|  | Miso, rice-koji miso, red type | 4.2 | 3.2 | < 0.001 |  |  |  |  |  |  |
|  | Miso, soybean-koji miso | 3.7 | 2.8 | < 0.001 |  |  |  |  |  |  |
|  | Cooking Sake, “Mitsukan Cooking Sake” | 1.7 | 1.2 | 0.002 |  |  |  |  |  |  |
| sweets, sugars & sweeteners | Traditional confectionery, "Mushi-manju" (steamed sweet dough stuffed with red bean paste) | 2.2 | 1.6 | 0.034 |  |  |  |  |  |  |
| green tea | Green tea, "Sencha" (common grade tea), infusion | 474.1 | 299.9 | < 0.001 |  |  |  |  |  |  |

| **Table 13**. The difference (95% CIs) in relative volumes of total gray matter and total white matter at baseline and follow-up across the three dietary patterns in men and women (n =1636) ^a, b^ | | | | | | | | |
| --- | --- | --- | --- | --- | --- | --- | --- | --- |
|  | | | | Total gray matter | |  | Total white matter | |
|  |  |  |  | *β* (95% CI) | *P*-value |  | *β* (95% CI) | *P*-value |
| Men (n = 815) | | | |  |  |  |  |  |
|  | Baseline | | |  |  |  |  |  |
|  |  | Model 1 ^c^ | |  |  |  |  |  |
|  |  |  | Vegetable-Fruit-Dairy diet | 2.3E-3 (-1.8E-3 to 6.4E-3) | 0.280 |  | -8.6E-4 (-4.8E-3 to 3.1E-3) | 0.672 |
|  |  |  | Traditional Japanese diet | -4.8E-5 (-3.9E-3 to 3.8E-3) | 0.980 |  | -4.4E-5 (-3.7E-3 to 3.6E-3) | 0.982 |
|  |  | Model 2 ^d^ | |  |  |  |  |  |
|  |  |  | Vegetable-Fruit-Dairy diet | 1.9E-3 (-2.2E-3 to 6.1E-3) | 0.355 |  | -7.6E-4 (-4.8E-3 to 3.3E-3) | 0.712 |
|  |  |  | Traditional Japanese diet | -1.1E-4 (-3.9E-3 to 3.7E-3) | 0.955 |  | 6.4E-5 (-3.6E-3 to 3.8E-3) | 0.973 |
|  | Follow-up | | |  |  |  |  |  |
|  |  | Model 1 ^c^ | |  |  |  |  |  |
|  |  |  | Vegetable-Fruit-Dairy diet | 3.2E-3 (-8.9E-4 to 7.3E-3) | 0.125 |  | -8.6E-4 (-4.8E-3 to 3.1E-3) | 0.674 |
|  |  |  | Traditional Japanese diet | 8.5E-4 (-3.0E-3 to 4.7E-3) | 0.662 |  | -9.0E-4 (-4.6E-3 to 2.8E-3) | 0.636 |
|  |  | Model 2 ^d^ | |  |  |  |  |  |
|  |  |  | Vegetable-Fruit-Dairy diet | 2.8E-3 (-1.4E-3 to 6.9E-3) | 0.189 |  | -5.7E-4 (-4.6E-3 to 3.5E-3) | 0.781 |
|  |  |  | Traditional Japanese diet | 7.1E-4 (-3.1E-3 to 4.5E-3) | 0.714 |  | -6.4E-4 (-4.4E-3 to 3.1E-3) | 0.736 |
|  |  |  |  |  |  |  |  |  |
| Women (n = 821) | | | |  |  |  |  |  |
|  | Baseline | | |  |  |  |  |  |
|  |  | Model 1 ^c^ | |  |  |  |  |  |
|  |  |  | Grain-Vegetable-Fruit diet | -3.4E-3 (-7.6E-3 to 7.2E-4) | 0.106 |  | -1.8E-3 (-5.7E-3 to 2.1E-3) | 0.373 |
|  |  |  | Traditional Japanese diet | 1.1E-4 (-3.3E-3 to 3.5E-3) | 0.950 |  | 7.0E-4 (-2.5E-3 to 3.9E-3) | 0.664 |
|  |  | Model 2 ^d^ | |  |  |  |  |  |
|  |  |  | Grain-Vegetable-Fruit diet | -3.7E-3 (-7.9E-3 to 4.6E-4) | 0.082 |  | -2.0E-3 (-5.9E-3 to 1.9E-3) | 0.317 |
|  |  |  | Traditional Japanese diet | 2.2E-4 (-3.2E-3 to 3.6E-3) | 0.900 |  | 8.8E-4 (-2.3E-3 to 4.0E-3) | 0.588 |
|  | Follow-up | | |  |  |  |  |  |
|  |  | Model 1 ^c^ | |  |  |  |  |  |
|  |  |  | Grain-Vegetable-Fruit diet | -3.2E-3 (-7.3E-3 to 9.0E-4) | 0.127 |  | -1.1E-3 (-5.0E-3 to 2.8E-3) | 0.568 |
|  |  |  | Traditional Japanese diet | 1.3E-3 (-2.0E-3 to 4.7E-3) | 0.438 |  | 2.1E-4 (-3.0E-3 to 3.4E-3) | 0.899 |
|  |  | Model 2 ^d^ | |  |  |  |  |  |
|  |  |  | Grain-Vegetable-Fruit diet | -3.5E-3 (-7.6E-3 to 6.3E-4) | 0.098 |  | -1.4E-3 (-5.3E-3 to 2.5E-3) | 0.493 |
|  |  |  | Traditional Japanese diet | 1.4E-3 (-2.0E-3 to 4.8E-3) | 0.414 |  | 3.8E-4 (-2.8E-3 to 3.6E-3) | 0.817 |
| a | Relative volume of region of interest (ROI) = ROI volume (mm^3^)/total intracranial volume (mm^3^) | | | | | | | |
| b | Analyzed by the general linear model. participants in the Western diet were used as the reference group. | | | | | | | |
| c | Adjusted for baseline information on age (years; continuous), APOE genotype (APOE-ε4 carriers: 2/4, 3/4, 4/4, or APOE-ε4 noncarriers: 2/2, 2/3, 3/3), education level (≤9, 10−12, or ≥13 years), smoking status (never, former, or current), total physical activity (METs-h/day; continuous), and energy intake (kcal/day; continuous). | | | | | | | |
| d | Adjusted for Model 1 plus baseline information on BMI (kg/m^2^; continuous), depressive symptoms (CES-D score; ≤15 or ≥16), and medical history (i.e., stroke, hypertension, heart disease, dyslipidemia, and diabetes; dichotomous yes or no responses, for each). | | | | | | | |

| **Table 14**. Baseline characteristics of individuals excluded from the study, categorized by sex (n = 666) | | | | | | | | |
| --- | --- | --- | --- | --- | --- | --- | --- | --- |
|  | | Total participants | | Men (n = 358) | | Women (n = 308) | | *P*-value ^a^ |
| Age (years); mean (SD) | | 65.5 (13.5) | | 64.3 (13.6) | | 66.9 (13.2) | | 0.012 |
| APOE-ε4 carriers ^b^; % | | 18.0 | | 19.8 | | 15.9 | | 0.225 |
| BMI (kg/m^2^); mean (SD) | | 22.9 (3.0) | | 23.0 (2.7) | | 22.7 (3.3) | | 0.120 |
| Medical history (yes); % | |  |  |  |  |  |  |  |
|  | Stroke | 7.4 | | 7.6 | | 7.1 | | 0.954 |
|  | Hypertension | 37.8 | | 39.9 | | 35.4 | | 0.259 |
|  | Heart disease | 10.8 | | 13.1 | | 8.1 | | 0.051 |
|  | Dyslipidemia | 21.2 | | 16.5 | | 26.6 | | 0.002 |
|  | Diabetes | 10.4 | | 11.5 | | 9.1 | | 0.385 |
|  | Dementia | 1.5 | | 0.8 | | 2.3 | | 0.200 |
| Current smoker; % | | 16.5 | | 26.5 | | 4.9 | | < 0.001 |
| Total physical activity (METs-h/day); mean (SD) | | 34.5 (4.1) | | 34.3 (4.9) | | 34.9 (2.9) | | 0.056 |
| Education level (years); % | |  |  |  |  |  |  |  |
|  | ≤9 | 27.3 | | 26.3 | | 28.6 | | 0.015 |
|  | 10−12 | 39.5 | | 35.8 | | 43.8 | |  |
|  | ≥13 | 33.2 | | 38.0 | | 27.6 | |  |
| Depressive symptoms ^c^; % | | 18.8 | | 18.7 | | 18.8 | | 1.000 |
| Energy intake (kcal/day); mean (SD) | | 1994.4 (490.1) | | 2241.3 (485.4) | | 1724.8 (326.2) | | < 0.001 |
| a | For continuous variables, the general linear model was used; for categorical variables, the χ^2^ test and Fisher's Exact Test were used. | | | | | | | |
| b | APOE genotype was defined as APOE-ε4 carriers (2/4, 3/4, 4/4) and APOE-ε4 noncarriers (2/2, 2/3, 3/3). | | | | | | | |
| c | Defined by the Center for Epidemiologic Studies Depression Scale (CES-D) score ≥16. | | | | | | | |
